# Unraveling the Stereoisomer Configurations of 1,1’‐bis(*tert*‐butylphosphino)Ferrocene in the Gas Phase

**DOI:** 10.1002/cphc.202400881

**Published:** 2024-12-15

**Authors:** Wenhao Sun, Denis Kargin, Zsolt Kelemen, Rudolf Pietschnig, Melanie Schnell

**Affiliations:** ^1^ Deutsches Elektronen-Synchrotron DESY Notkestr. 85 22607 Hamburg Germany; ^2^ Institute of Chemistry and CINSaT University of Kassel Heinrich-Plett-Str. 40 34132 Kassel Germany; ^3^ Department of Inorganic and Analytical Chemistry Budapest University of Technology and Economics Műegyetem Rkp 3. 1111 Budapest Hungary; ^4^ Institute of Physical Chemistry Christian-Albrechts-Universität zu Kiel Max-Eyth-Str. 1 24118 Kiel Germany

**Keywords:** Ferrocene derivative, Bisphosphines, Stereochemistry, Microwave spectroscopy, Dispersion stabilization

## Abstract

The molecular structure of a ferrocene derivative with adjacent centers of chirality, 1,1’‐bis(*tert*‐butylphosphino)ferrocene, has been examined in the gas phase using broadband microwave spectroscopy under the isolated and cold conditions of a supersonic jet. The diastereomers of 1,1’‐bis(*tert*‐butylphosphino)ferrocene can adopt homo‐ and hetero‐chiral configurations, owing to the P‐chiral substituents on the cyclopentadienyl rings. Moreover, the internal ring rotation of each diastereomer gives rise to four conformers with eclipsed ring arrangements, where the two *tert*‐butylphosphino groups were separated by dihedral angles of approximately 72°, 144°, 216°, and 288° with respect to the two ring centers. The interconversion barriers between the conformations are below 2 kJ/mol, whereas the pyramidal inversion of the *tert*‐butylphosphino groups is hindered by more than 140 kJ/mol, calculated at the B3LYP−D3(BJ)/def2‐QZVP level of theory. In the experimental microwave spectrum, we unambiguously identified the two global‐minimum diastereomers with 72° conformations. The absence of other conformers can be attributed to the relaxation dynamics in the supersonic jet, which transfers the high‐energy conformers to the respective global‐minimum geometries. Additionally, we discovered that London dispersion interactions between the two *tert*‐butylphosphino groups play a crucial role in stabilizing the structures of this ferrocene complex.

## Graphical abstract

1,1’‐Bis(*tert*‐butylphosphino)ferrocene features exceptional conformational flexibility along with stable P‐stereogenic centers, making it an intriguing organometallic compound for asymmetric catalysis. By combining microwave spectroscopy with theoretical calculations, we characterized its stereoisomeric configurations under isolated conditions, providing deeper insights into its structural dynamics.

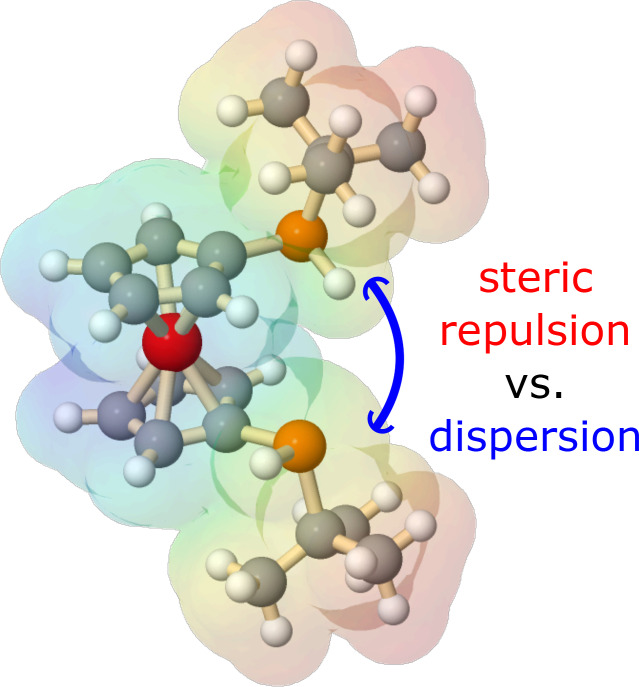

## Introduction

**Figure 1:**
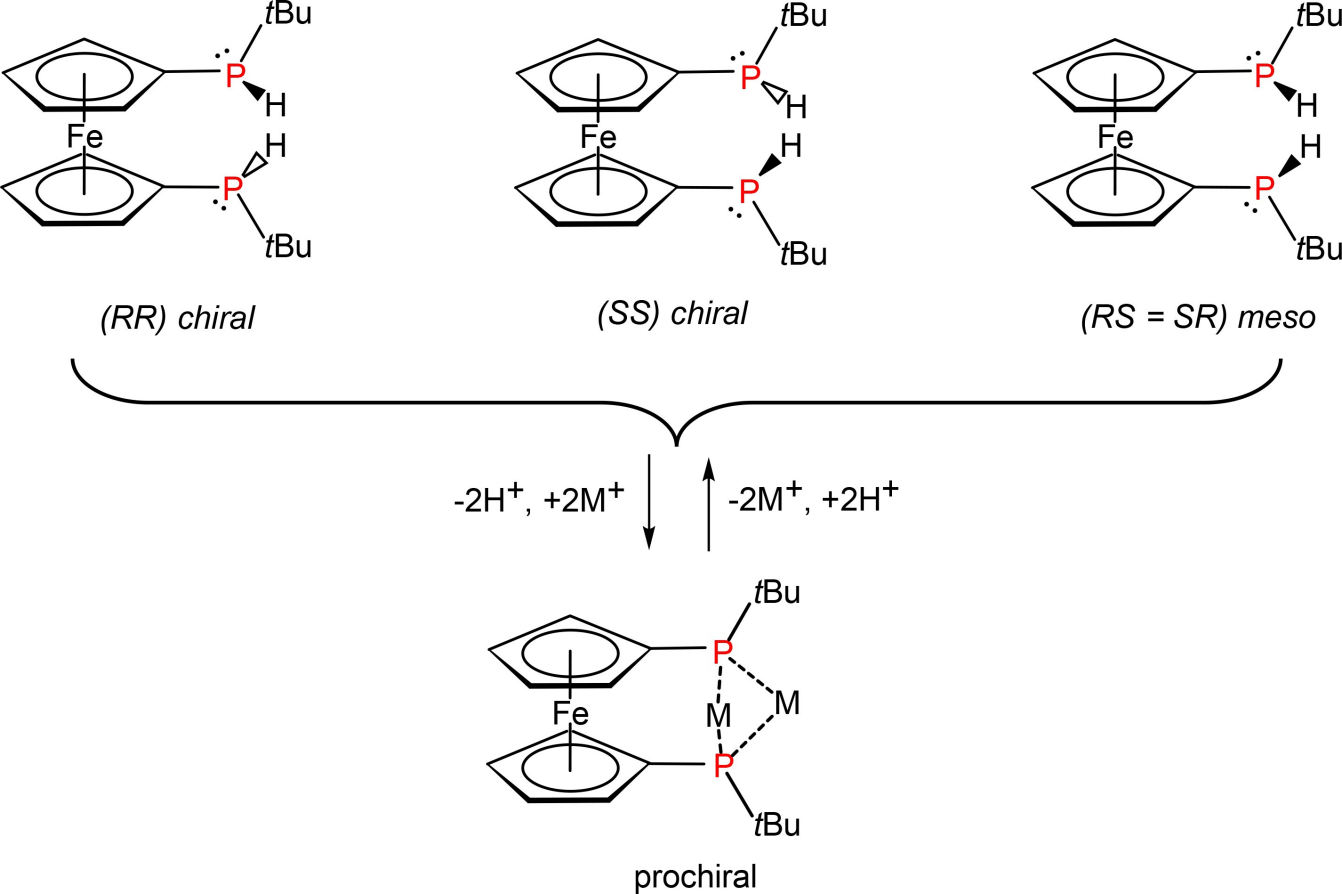
Epimerization of 1,1’‐bis(*tert*‐butylphosphino)ferrocene via proton abstraction and re‐addition. The homo‐chiral configurations (*RR* and *SS*) are optically active, with the hetero‐chiral or *meso* configuration (*RS*) being optically inactive. The *tert*‐butyl groups provide steric protection, stabilizing the compound during reactions.

Bisphosphines, sometimes called diphosphines and according to IUPAC preferably named bisphosphanes,^[1]^ are compounds of fundamental importance with widespread applications, for instance, as bidentate ligands in transition metal complexes and catalysts.^[2]^ Ferrocene bridged bisphosphines feature an electron rich metallocene unit and have been varied with respect to their substitution pattern and steric congestion.^[3]^ Unsymmetrically substituted bisphosphines contain P‐stereogenic centers entailing the presence of two diastereomers in a statistical mixture, i. e., the racemic mixture of the homo‐chiral forms (*RR* and *SS*) in addition to the hetero‐chiral meso‐forms (*RS* and *SR*) (Figure 1). The stereochemical integrity of P‐stereogenic phosphines may be compromised by several epimerization processes such as planar inversion[^4^, ^5^] or abstraction and subsequent re‐addition of electrophilic or nucleophilic substituents to a prochiral intermediate.^[6]^ The latter has been demonstrated for the secondary phosphine 1,1’‐bis(*tert*‐butylphosphino)ferrocene (BPF) in solution, where deprotonation leads to a single twofold symmetrically bridged prochiral product which has been isolated (Figure 1).[^7^, ^8^, ^9^, ^10^] Addition of electrophiles such as H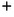 , halosilanes, halophosphines and other element halides results in formation of a statistical mixture of *rac* and *meso* diastereomers.^[7]^ Interestingly, bifunctional electrophiles may bridge both phosphorus atoms, and the resulting ring formation of the achiral meso isomeric form is observed exclusively,[^11^, ^12^, ^13^, ^14^, ^15^, ^16^, ^17^, ^18^] aside from a few exceptions.^[19]^ Intramolecular donor interactions of two stereogenic phosphanyl units via prochiral transition states or intermediates, as in the case of the lithiated BPF, have been investigated in solution and the solid state.[^7^, ^9^]

Regarding the unbridged 1,1’‐disubstituted ferrocene complexes including BPF, they exhibit high conformational flexibility, which inherently influences their chemical reactivity and stability. In solution and the gas phase, the two cyclopentadienyl (Cp) rings in such 1,1’‐disubstituted ferrocene derivatives commonly adopt the nearly eclipsed arrangements, with the staggered conformations being the transition states.^[20]^ For the minimum‐energy geometries, the inter‐ring torsional angles (ϕ , the dihedral angle between the two ring substituents relative to the center of the Cp rings) are approximately 0° (synperiplanar), 72°/288° (synclinal), and 144°/216° (anticlinal).^[21]^ The interconversion barriers between them are typically within a few kcal/mol, with the unsubstituted ferrocene featuring a barrier of 0.9(3) kcal/mol.^[22]^ This torsional barrier is sensitive to the interplay between the two substituent groups on the Cp ligands and the surrounding chemical environments. With the increasing size of the substituent groups, the 0° eclipsed conformation may turn into an energy maximum due to the enhanced steric repulsion. In addition, as the barriers of the internal ring rotation can be readily surmounted, their solid‐state structures may adopt different arrangements due to crystal packing effects. For instance, in the gas phase, 1,1’‐di‐*tert*‐butylferrocene is characterized with eclipsed configurations ($C_{2}$ molecular symmetry), where the ring torsional angles (ϕ ) are approximately 72° and 144°. However, in the solid state, it exhibits a staggered $C_{2h}$ symmetry with a torsional angle ϕ of 180°, even though this conformation represents a transition state in the gas phase.^[20]^

In this respect, microwave spectroscopy offers a valuable tool for exploring the conformational behaviors of ferrocene compounds with great sensitivity to isomers and conformers. Its capability to characterize gas‐phase molecules in isolation allows for detailed investigation free from the influence of crystal packing effects. Although ferrocene itself is non‐polar and thus transparent to microwave radiation, various ferrocene systems, including monosubstituted ferrocene derivatives and ferrocene‐water complexes, have been successfully characterized using microwave spectroscopy.[^23^, ^24^, ^25^, ^26^, ^27^, ^28^, ^29^] 1,1’‐Dimethylferrocene remains the only example of a 1,1’‐disubstituted ferrocene derivative studied by this method so far.^[21]^ With the substitution of two methyl groups, two conformers (ϕ =0° and 72°) were unambiguously identified in the rotational spectrum, while solely the 0° conformation was observed in the solid state by X‐ray crystallography.^[30]^

Herein, we report the high‐resolution microwave spectroscopic measurements of 1,1’‐bis(*tert*‐butylphosphino)ferrocene in the gas phase. Based on the experimental findings, we explored, in more detail, the conformational landscape arising from the inter‐ring rotation and the diastereomerism due to the two P‐stereogenic centers, which entail two diastereomers. The spectral analysis and understanding of the conformational dynamics are supported by theoretical calculations.

## Experimental Details

The title compound, 1,1’‐bis(*tert*‐butylphosphino)ferrocene (BPF), was prepared according to a published procedure (see Supplementary Information), which was a statistical mixture of two diastereomers.^[7]^ Identity and purity have been established with 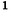 H, 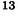 C and 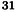 P NMR spectroscopy in solution using Jeol HNM‐ECZL500, Varian V NMRS‐500 MHz or Mr‐400 MHz spectrometers at 25 °C. Chemical shifts were referenced to residual protic impurities in the solvent (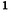 H) or the deuterio solvent (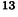 C) and reported relative to external H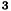 PO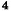 (85 %) (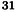 P). The yielded compound exhibits good air stability, which is a consequence of the protective properties of ferrocene as highlighted only very recently.^[31]^

In this work, the microwave spectroscopy was carried out in the frequency range of 2–8 GHz, using the chirped pulse Fourier transform microwave (CP‐FTMW) spectrometer COMPACT. The operation principles have been described in detail elsewhere.^[32]^ The crystalline sample was held in a homemade, heatable reservoir close to the solenoid valve (General valve Series 9). The sample was maintained at 170 °C to generate sufficient vapor pressure, which was diluted into neon carrier gas at a stagnation pressure of approximately 1 bar. This backing pressure of neon was found to be optimal, which is consistent with previous microwave studies of other ferrocene derivatives.[^21^, ^23^, ^24^, ^25^, ^26^, ^27^, ^28^]

The gas mixture was supersonically expanded into the vacuum chamber via the solenoid valve operated at 8 Hz. Molecules in the supersonic jet were polarized by a chirped microwave pulse eight times, resulting in an overall repetition rate of 64 Hz. After each interaction, the decay of the macroscopic polarization of the molecular ensemble in the jet was recorded as a function of time, known as free induction decay (FID). A total of 6 × 10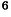 FID acquisitions were collected and averaged on a fast oscilloscope, and fast Fourier transformed (FFT) to obtain the frequency‐domain spectrum. The full‐width‐at‐half‐maximum (FWHM) line width in the spectrum is about 60 kHz and the frequency accuracy is about 10 kHz.

### Theoretical Calculations

The conformational searches of homo‐ and hetero‐chiral BPF were explored individually with the CREST program using the GFN2‐xTB method.[^33^, ^34^] The generated conformational structures were further optimized at the B3LYP/def2‐QZVP level of theory coupled with D3(BJ) dispersion corrections using the ORCA program package, version 5.0.4.[^35^, ^36^, ^37^] Harmonic frequency calculations were performed to confirm real energy minima and to provide vibrational zero‐point energy (ZPE) corrections. Following the B3LYP−D3(BJ) calculations, single‐point calculations were conducted on the obtained minimum‐energy structures using the linear‐scaling local natural orbital coupled‐cluster (LNO‐CCSD(T)) method with the def2‐QZVPP basis set, as implemented in MRCC.[^38^, ^39^]

To achieve a comprehensive understanding of the conformational landscape, the torsional potential energy of the ring rotation was scanned as a function of the relative dihedral angle (ϕ ) of the two *tert*‐butylphosphino groups in steps of 6°, while two dummy atoms were positioned at the centers of the Cp rings to establish the rotation axis (see Figure S2 in the Supplementary Information). The transition states connecting the energy minima were fully optimized and characterized with one imaginary frequency. Moreover, the pyramidal inversion motion of the *tert*‐butylphosphino group was calculated using the nudged elastic band (NEB) method.^[40]^ Both calculations were performed at the B3LYP−D3(BJ)/def2‐QZVP level of theory using the ORCA program.

## Results and Discussion

**Figure 2:**
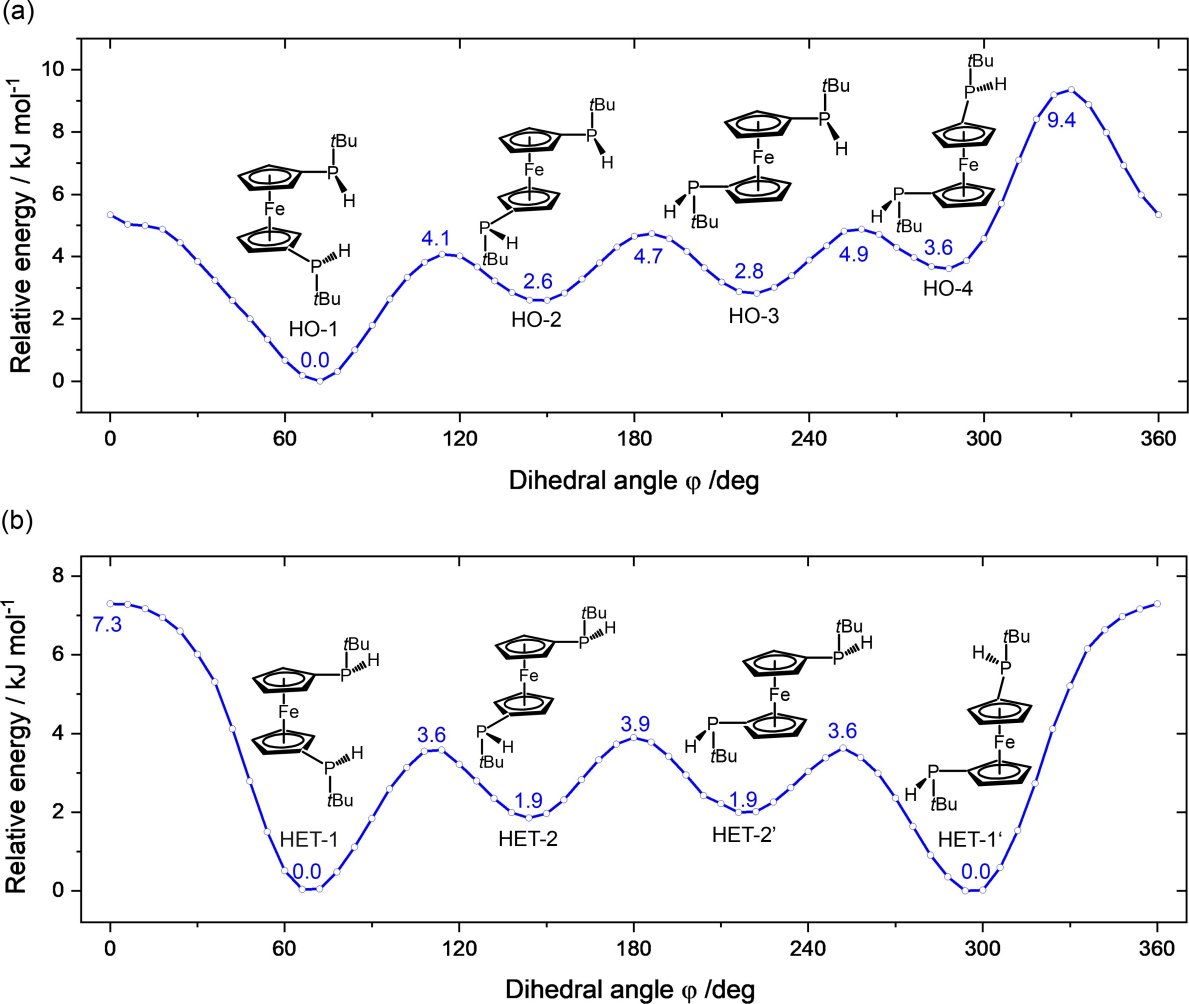
Torsional potential energy curves for the internal ring rotations of 1,1’‐bis(*tert*‐butylphosphino)ferrocene in its homo‐chiral (a) and hetero‐chiral (b) configurations, calculated at the B3LYP−D3(BJ)/def2‐QZVP level of theory. The torsional angle (ϕ ) is defined by the relative dihedral angle between the two *tert*‐butylphosphino groups with respect to the two ring centers. HET‐1/1’ and HET‐2/2’ represent two pairs of enantiomers that produce identical microwave spectra. Note that the energies of HO‐1 and HET‐1 are both set to 0 kJ/mol for convenience, of which HO‐1 is 0.7 kJ/mol more stable. The relative energies of the minimum geometries are provided in Table 1.

In 1,1’‐bis(*tert*‐butylphosphino)ferrocene (BPF), the pyramidal nature of the phosphorus atoms leads to P‐stereogenic *tert*‐butylphosphino groups. Their substitutions on the Cp rings give rise to the homo‐chiral (*RR* and *SS*) and hetero‐chiral (*RS* and *SR*) configurations of BPF. Both diastereomers were characterized in solution by NMR spectroscopy, with a ratio of approximately 1 : 1.^[7]^ According to theoretical calculations, in the energetically low‐lying isomers, the two *tert*‐butyl groups are favorably opposing each other, directed away from the transition‐metal center to reduce steric repulsion. The conformational landscapes regarding the internal ring rotation of the homo‐ and hetero‐chiral BPF diastereomers are demonstrated by the potential energy curves (PECs), as shown in Figure 2. The corresponding interconversion pathways, including zero‐point energy corrections, are provided in Supplementary Figure S3.

Theoretically, there are four conformers predicted for the homo‐chiral diastereomers. In all of them, the Cp rings adopt the eclipsed arrangements, where the two *tert*‐butyl‐phosphino groups exhibit dihedral angles (ϕ ) of approximately 72° (HO‐1, eclipsed synclinal), 150° (HO‐2, eclipsed anticlinal), 222° (HO‐3, eclipsed anticlinal), and 288° (HO‐4, eclipsed synclinal), respectively. Due to the steric repulsion, the eclipsed synperiplanar configuration ($\phi =0^{\circ }$ ), which is preferred in some other 1,1’‐disubstituted ferrocenes,[^20^, ^21^] is not a local energy minimum in this case. In terms of hetero‐chiral BPF, four local energy minima were revealed on the torsional potential energy profile as well. However, they correspond to two pairs of equivalent configurations. The eclipsed synclinal conformers ($\phi =66^{\circ }$ and 294°) and the eclipsed anticlinal conformers ($\phi =144^{\circ }$ and 216°) are two pairs of enantiomers, denoted as HET‐1/HET‐1’ and HET‐2/HET‐2’.

The total of six diastereomers of homo‐ and hetero‐chiral BPF were further optimized at the B3LYP−D3(BJ) level of theory without structural constraints, and the obtained theoretical spectroscopic parameters are provided in Table 1, including relative energies, rotational constants ($A_{e}$ , $B_{e}$ , and $C_{e}$ ) of the equilibrium geometries and the associated dipole‐moment components ($|\mu_{a}|$ , $|\mu_{b}|$ , and $|\mu_{c}|$ ) along the *a*‐, *b*‐, and *c*‐axes in the principal axis system. In both cases, the eclipsed synclinal arrangements (HO‐1 and HET‐1) are the preferred configurations, with HO‐1 being the global minimum isomer, which is 0.7 kJ/mol more stable than HET‐1 after vibrational zero‐point energy (ZPE) corrections. The relative energies of these diastereomers were also estimated using the single‐point energy calculations at the LNO‐CCSD(T)/def2‐QZVPP, ω B97X−D/def2‐QZVPP, and M06‐2X/def2‐QZVPP levels of theory. The results are generally consistent with the B3LYP−D3(BJ) calculations, with deviations well within the chemical accuracy of these methods (see Table 1 and supplementary Table S1). A comparison between the B3LYP−D3(BJ) and LNO‐CCSD(T) energies suggests that the D3(BJ) corrections may slightly overestimate the dispersion forces.

**Table 1:** Theoretical molecular properties for the diastereomers of 1,1’‐bis(*tert*‐butylphosphino)ferrocene, calculated at the B3LYP−D3(BJ)/def2‐QZVP level of theory.

Isomers	ϕ ^*a*^/ 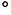	$\Delta E_{B3}$ ^*b*^	$\Delta E_{\mathrm{LNO}}$ ^*c*^	$A_{e}$ /MHz	$B_{e}$ /MHz	$C_{e}$ /MHz	$|\mu_{a}|$ /D	$|\mu_{b}|$ /D	$|\mu_{c}|$ /D
Homo‐chiral stereoisomers									
HO‐1	68.9	0.0	0.0	366.2	176.8	139.6	0.0	0.8	0.0
HO‐2	146.9	2.4	1.2	662.5	120.0	119.3	0.0	0.0	0.4
HO‐3	218.9	2.6	1.1	719.4	118.5	116.3	0.0	0.0	1.4
HO‐4	284.7	3.2	2.5	425.0	154.5	133.0	0.0	1.7	0.0

Hetero‐chiral stereoisomers									
HET‐1/HET‐1‘	66.3/293.7	0.7	0.9	376.9	172.4	138.5	0.6	1.2	0.8
HET‐2/HET‐2‘	148.8/211.2	2.5	0.9	666.2	120.0	119.3	0.3	0.1	0.6

^*a*^ Dihedral angles between the two phosphorus atoms with respect to the centers of the two cyclopentadienyl rings. ^*b*^ Relative energies (in kJ/mol) calculated at the B3LYP−D3(BJ)/def2‐QZVP level of theory, with vibrational zero‐point energy corrections. ^*c*^ Relative energies (in kJ/mol) obtained with single‐point energy calculations at the LNO‐CCSD(T)/def2‐QZVPP level of theory, using optimized structures from B3LYP−D3(BJ) calculations.

**Figure 3:**
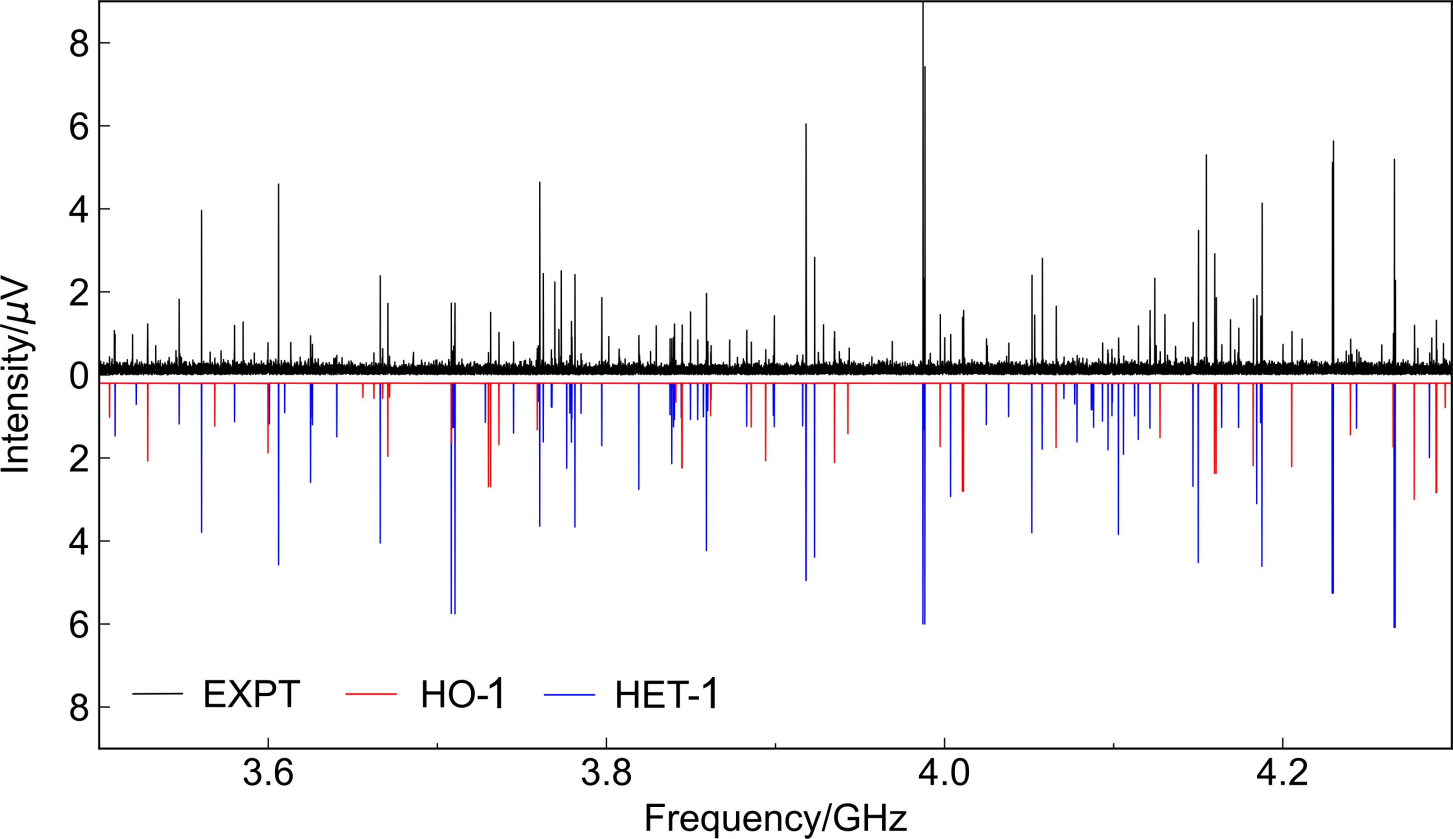
Portion of the microwave spectrum measured with 1,1’‐bis(*tert*‐butylphosphino)ferrocene from 3.5 to 4.3 GHz. It consists of an average of 6.0 × 10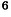 FID acquisitions. The experimental spectrum (EXPT) is shown in the upper trace. The simulated spectra of the HO‐1 and HET‐1 diastereomers at a rotational temperature of 1 K are displayed in the bottom trace, which are based on the fitted rotational constants provided in Table 2.

In the experiment, two diastereomers were identified in the microwave spectrum, as shown in Figure 3. The two distinguished rotational fingerprints are individually fitted with the SPFIT program, using Watson's *A*‐reduced Hamiltonian in the I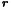 representation,[^41^, ^42^, ^43^] and the resulting spectroscopic parameters are summarized in Table 2. By comparing the experimentally determined ground‐state rotational constants ($A_{0}$ , $B_{0}$ , and $C_{0}$ ) with theoretical values ($A_{e}$ , $B_{e}$ , and $C_{e}$ ), it is evident that these two rotational spectra correspond to HO‐1 and HET‐1, while there is clearly no agreement with the other conformations. The deviations between experimental and theoretical rotational constants are better than 2.2 %. The centrifugal distortion constants ($\Delta_{J}$ , $\Delta_{JK}$ , $\Delta_{K}$ , $\delta_{J}$ , and $\delta_{K}$ ) are also calculated based on the harmonic frequency calculations using a home‐written python script.^[41]^ The results show satisfactory consistency with the corresponding fits.

**Table 2:** Experimental spectroscopic parameters of the HO‐1 and HET‐1 diastereomers obtained from SPFIT least‐squares fits, in comparison with the theoretical results obtained at the B3LYP‐D3(BJ)/def2‐QZVP level of theory.

Parameters^*a*^	HO‐1 ( 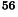 Fe)		HET‐1 ( 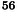 Fe)		HET‐1 ( 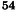 Fe)
	theo.	expt.	theo.	expt.	expt.
A /MHz	366.2	374.52123(11)	376.9	383.343772(73)	384.51987(10)
B /MHz	176.8	174.78454(10)	172.4	171.744856(39)	171.74427(25)
C /MHz	139.6	140.013339(53)	138.5	139.230186(30)	139.384694(87)
$\Delta_{J}$ ^*b*^/kHz	0.0104	0.01108(20)	0.0147	0.013359(59)	[0.013359]^*c*^
$\Delta_{JK}$ /kHz	−0.0583	−0.06691(84)	–0.0929	−0.08923(32)	[−0.08923]
$\Delta_{K}$ /kHz	0.1139	0.1375(10)	0.1899	0.18977(43)	[0.18977]
$\delta_{J}$ /kHz	0.0040	0.00421(10)	0.0058	0.005167(29)	[0.005167]
$\delta_{K}$ /kHz	0.0065	0.0088(23)	0.0133	0.01363(71)	[0.01363]
N (a|b|c )^*d*^		208 (0| 208| 0)		683 (150| 400| 133)	54 (0| 54| 0)
RMS ^*e*^/kHz		4.7		6.4	5.9

^*a*^ Fitted with Watson's *A*‐reduced Hamiltonian in its I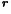 representation using Pickett's SPFIT program. ^*b*^ Centrifugal distortion constants were calculated from the harmonic frequency calculations using a home‐written python script. ^*c*^ Values in box brackets were fixed. ^*d*^ Total number (N) of lines in the fit and the number of lines for each type of rotational transitions (*a*‐, *b*‐, and *c*‐type). ^*e*^ Root‐mean‐square deviation of the fit, RMS=$\sqrt{\frac{\sum {\left(\nu_{obs}-\nu_{calc}\right)}^{2}}{N}}$ .

Moreover, the spectral assignments are supported by the calculated dipole‐moment components. For HO‐1, it possesses a $C_{2}$ molecular symmetry, where the $C_{2}$ rotation axis is aligned with its principal inertia *b*‐axis. This cancels out the dipole‐moment component along the *a*‐ and *c*‐axes ($\mu_{a}$ and $\mu_{c}$ ), leaving a nonzero $\mu_{b}$ component of about 0.8 D. In the corresponding rotational spectrum (red trace in Figure 3), solely *b*‐type transitions were detected, matching with its dipole‐moment orientation. A total of 208 *b*‐type transition frequencies were included in the least‐squares fit, leading to an overall root‐mean‐square (RMS) deviation of about 4.7 kHz. On the other hand, the geometry of HET‐1 belongs to the $C_{1}$ point group. The dipole‐moment components are predicted to be $|\mu_{a}|$ =0.6 D, $|\mu_{b}|$ =1.2 D, and $|\mu_{c}|$ =0.8 D. As shown in Table 2, all three types (*a*‐, *b*‐, and *c*‐type) of rotational transitions were observed in its rotational spectrum. The relative intensity ratios of the *a*‐, *b*‐ and *c*‐type rotational transitions are consistent with the calculated dipole‐moment components (blue trace in Figure 3). A total of 683 transition frequencies were measured, giving an RMS deviation of about 6.4 kHz in the fit. Both the well‐predictable centrifugal distortion constants and the small RMS deviations of the least‐squares fits indicate the rigidity of the molecular structures in a jet‐cooled collision‐free environment.

Although HO‐1 and HET‐1 are nearly isoenergetic, the overall spectrum of HO‐1 is lower in intensity compared to that of HET‐1 due to its smaller dipole moment. By quantifying the relative intensities of the *b*‐type transitions from both diastereomers with the associated computed $\mu_{b}$ components, their relative abundance in the gas jet is estimated to be about 50 % : 50 %, matching the solution‐phase NMR results. In Figure 3, the simulated spectra of these two species were plotted with a 1 : 1 abundance ratio, which is comparable with the experimental trace. Given the good signal‐to‐noise ratio (SNR) of the HET‐1 spectrum (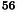 Fe), its 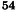 Fe singly substituted isotopologue was observed in its natural abundance (5.9 %). The rotational constants were experimentally fitted with 54 identified transition frequencies, giving an overall RMS deviation of 5.9 kHz, while the centrifugal distortion constants were fixed at the values of the normal species.

**Figure 4:**
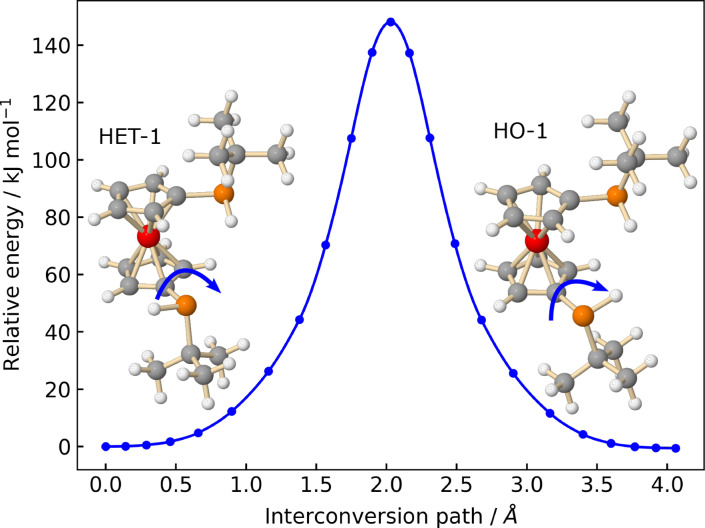
Energy minimum path of the pyramidal inversion of the *tert*‐butylphosphino group in BPF, calculated using the nudged elastic band (NEB) method at the B3LYP−D3(BJ)/def2‐QZVP level of theory. The x‐axis describes the structural displacements (in Å) along the inversion motion path. The arrows indicate the motion of the hydrogen atom in the pyramidal inversion of the *tert*‐butylphosphino group.

Regarding the non‐observed conformations, it is likely that they underwent relaxation toward HO‐1 and HET‐1, respectively, in the supersonic jet. Because the interconversion barriers are within 2 kJ/mol, as can be seen in Figure 2 and Supplementary Figure S3, well below the conformational relaxation threshold of approximately 4.8 kJ/mol.^[44]^ Additionally, we explored the interconversion pathway between the HO‐1 and HET‐1 diastereomers, which are linked by the pyramidal inversion motion of the *tert*‐butylphosphino group, also known as the ‘umbrella’ motion. Such motion in secondary phosphines goes through an sp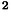 planar transition state at phosphorus with a typical barrier of ∼ 30–35 kcal/mol.^[45]^ The calculation was carried out with the NEB method at the B3LYP−D3(BJ)/def2‐QZVP level of theory.^[40]^ With given starting and ending geometries, namely HET‐1 and HO‐1, the NEB algorithm automatically samples a series of structural images in between, and optimizes and minimizes the interaction forces between the adjacent structures to find the minimum energy path and the saddle point. As shown in Figure 4, the barrier of the pyramidal inversion at phosphorus is about 143.5 kJ/mol (34.3 kcal/mol). Therefore, the P‐stereogenic centers in *tert*‐butylphosphino groups are quite stable, allowing for the detection of both homo‐ and hetero‐chiral BPF by both gas‐phase microwave spectroscopy and solution‐phase NMR spectroscopy.

**Figure 5:**
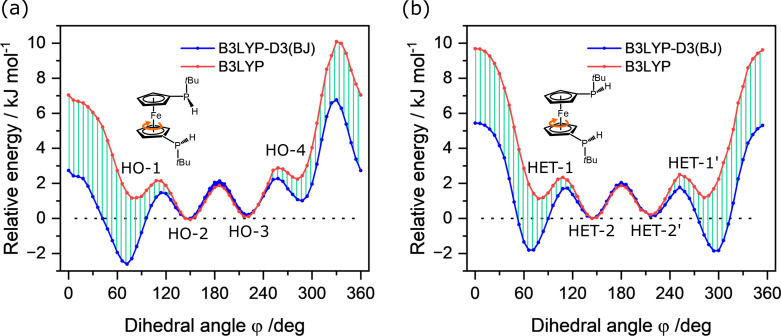
Torsional potential energy curves for homo (a) and hetero (b) chiral BPF, calculated at the B3LYP/def2‐QZVP level of theory with and without D3(BJ) dispersion corrections. The energies at 144° are set to 0 kJ/mol for comparison. The drop lines in green indicate the relative strength of the stabilizing dispersive interactions, quantified by the D3(BJ) corrections.

Furthermore, the conformation stability can be rationalized by interactions between the two *tert*‐butylphosphino groups. When the dihedral angles ϕ between these groups are less than $60^{\circ }$ , significant steric repulsion occurs, where the dispersion stabilization forces are not sufficient to compensate, as shown in Figure 5. Consequently, geometries at $\phi =0^{\circ }$ do not represent minimum‐energy configurations. Nevertheless for 1,1’‐disubstituted ferrocenes with smaller ring substituents, such as 1,1’‐dimethylferrocene, the $0^{\circ }$ conformation can be stably observed in the gas phase as well as in the solid phase.[^21^, ^30^] Additionally, for homo‐chiral BPF, the lone pair‐lone pair repulsion is enhanced when the two lone pairs on phosphorus are in close proximity, lifting the energy of HO‐4 higher than HO‐1 by 3.2 kJ/mol and resulting in an energy maximum around $330^{\circ }$ instead of $0^{\circ }$ . When the dihedral angles exceed $60^{\circ }$ , the steric repulsion decreases and London dispersion effects start to dominate. This effect can be roughly quantified by the D3(BJ) correction term in the DFT calculations (Figure 5), which has been validated in other studies.[^36^, ^46^, ^47^] As investigated in this work, without D3(BJ) corrections, the eclipsed anticlinal HO‐2/3 and HET‐2 conformers are predicted to be more stable than the eclipsed synclinal HO‐1 and HET‐1 conformations by approximately 1.2 kJ/mol. For HO‐1 and HET‐1, the dispersion stabilization effects are about 4 kJ/mol greater than that for HO‐2/3 and HET‐2, making HO‐1 and HET‐1 the global minima for homo‐ and hetero‐chiral arrangements, which agrees with our observations in the microwave spectrum. This evidently shows that owing to the facile internal ring rotation in ferrocene, not only the steric repulsion between the ring substituents but also the dispersion attraction forces can substantially influence the conformational arrangements.

In summary, we explored the gas‐phase conformations of 1,1’‐bis(*tert*‐butylphosphino)ferrocene using broadband microwave spectroscopy in combination with quantum chemical calculations. In the rotational spectrum, we identified two diastereomers with homo‐ and hetero‐chiral arrangements at the P‐stereogenic centers. The theoretical potential energy curves suggest that the eclipsed ring conformations, except for the $0^{\circ }$ conformation, are energy minima, while the staggered ring conformations serve as transition states. The internal ring torsional barriers are well below 2 kJ/mol, allowing facile transition to the global minimum geometries in the supersonic jet. Experimentally, the detected diastereomers indeed correspond to the global‐minimum geometries of the homo‐ and hetero‐chiral 1,1’‐bis(*tert*‐butylphosphino)ferrocene, where the two *tert*‐butyl‐ phosphino groups are separated by dihedral angles of approximately 66°. The conformational stability relies on a delicate balance between the steric repulsion and London dispersion attraction effects between the two *tert*‐butylphos‐phino groups.

## Conflict of Interests

There is no conflict of interest to declare.

1

## Supporting information

As a service to our authors and readers, this journal provides supporting information supplied by the authors. Such materials are peer reviewed and may be re‐organized for online delivery, but are not copy‐edited or typeset. Technical support issues arising from supporting information (other than missing files) should be addressed to the authors.

Supporting Information

## Data Availability

The data that support the findings of this study are available from the corresponding author upon reasonable request.
