# Supplementary material for: Unraveling the Stereoisomer Configurations of 1,1’‐bis(tert‐butylphosphino)Ferrocene in the Gas Phase
Source: Chemphyschem. 2024 Dec 15;26(5):e202400881. doi: 10.1002/cphc.202400881 (PMC11878755; doi:10.1002/cphc.202400881)
Supplement: Supplementary file 1 — Supporting Information [file CPHC-26-e202400881-s001.pdf]

ChemPhysChem

Supporting Information

Unraveling the Stereoisomer Configurations of 1,1′-bis(*tert*-butylphosphino)Ferrocene in the Gas Phase

Wenhao Sun, Denis Kargin, Zsolt Kelemen, Rudolf Pietschnig, and Melanie Schnell*

Supplementary information:

Unraveling the stereoisomer configurations of 1,1'-bis(tert-butylphosphino)ferrocene in the gas phase

Wenhao Sun,† Denis Kargin,‡ Zsolt Kelemen,¶ Rudolf Pietschnig,‡ and Melanie Schnell*,†,§

†*Deutsches Elektronen-Synchrotron DESY, Notkestr. 85, 22607 Hamburg, Germany*

‡*Institute of Chemistry and CINSaT, University of Kassel, Heinrich-Plett-Str. 40, 34132 Kassel, Germany*

¶*Department of Inorganic and Analytical Chemistry, Budapest University of Technology and Economics, 1111 Budapest, Hungary*

§*Institute of Physical Chemistry, Christian-Albrechts-Universität zu Kiel, Max-Eyth-Str. 1, 24118 Kiel, Germany*

E-mail: melanie.schnell@desy.de

Contents

1 Supplementary experimental details. **S1**

2 Supplementary theoretical details. **S2**

3 Cartesian coordinates of molecular geometries. **S3**

3.1 Cartesian coordinates of the homo-chiral conformers. S3

3.2 Cartesian coordinates of the hetero-chiral conformers. S11

4 Measured rotational transitions. **S16**

1 Supplementary experimental details.

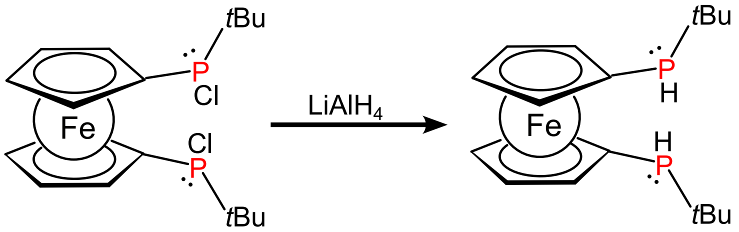

1 **2**

Figure S1: Preparation of 1,1'-bis(*tert*-butylphosphino)ferrocene.

A solution of **1** (7.26g, 16.8 mmol) in 10 mL Et_2O was added to a suspension of $LiAlH_4$ (637 mg, 16.8 mmol) in 20 mL of Et_2O. The reaction mixture was stirred for 15 minutes, and degassed water was added until the evolution of gas stopped. The residue was extracted with Et_2O, and the organic layer was washed with water and brine. The organic layer was dried with $MgSO_4$, and the solvent was removed *in vacuo*. Recrystallization from pentane yielded 85% product (5.15g, 14.2 mmol) as orange crystals.[1]

(1) Kargin, D., Kelemen, Z., Krekić, K., Maurer, M., Bruhn, C., Nyulászi, L. & Pietschnig, R. [3]Ferrocenophanes with the Bisphosphanotetryl Bridge: Inorganic Rings on the Way to Tetrylenes. *Dalton Trans.*. **45**, 2180-2189 (2016)

2 Supplementary theoretical details.

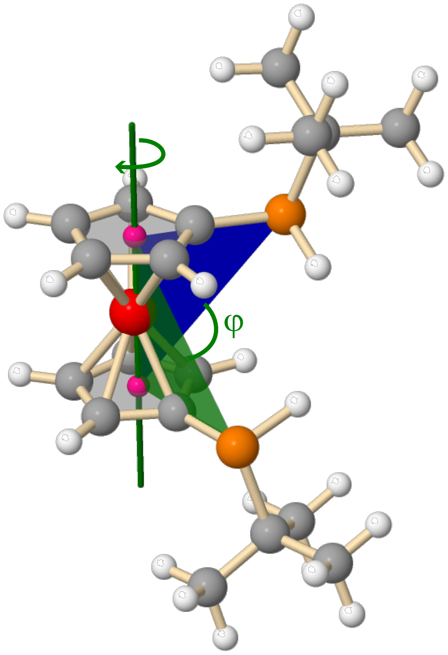

Figure S2: Dihedral angle φ of the internal ring rotation of 1,1'-bis(*tert*-butylphosphino)ferrocene. Two dummy atoms (X) are positioned at the centers of the cyclopentadienyl rings to indicate the rotation axis and φ = P-X-X-P.

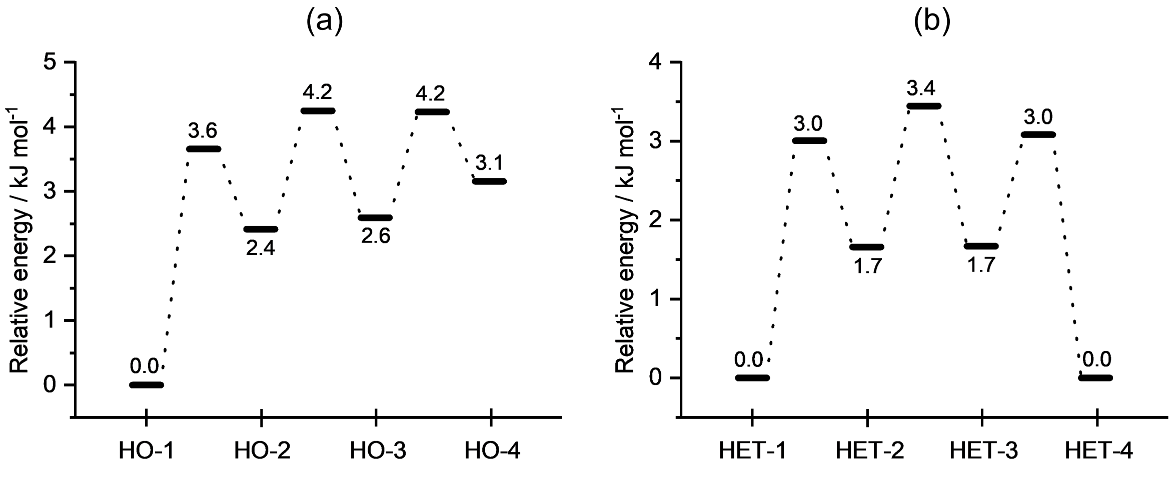

Figure S3: Zero-point energy corrected interconversion pathways for the four rotamers of homo-chiral (a) and hetero-chiral (b) 1,1'-bis(*tert*-butylphosphino)ferrocene, respectively.

Table S1: Relative energies (in kJ/mol) of the diastereomers of 1,1'-bis(*tert*-butylphosphino)ferrocene. Note that the B3LYP-D3(BJ) results are from structural optimizations, while the other three sets are obtained from single-point energy calculations using the B3LYP-D3(BJ) optimized structures.

	B3LYP-D3(BJ) def2-QZVP	LNO-CCSD(T) def2-QZVPP	ωB97X-D def2-QZVPP	M06-2X def2-QZVPP
homo-chiral stereoisomers				
HO-1	0	0.0	0.0	0.0
HO-2	2.6	1.2	1.8	1.0
HO-3	2.8	1.1	2.2	1.2
HO-4	3.6	2.5	3.4	2.4
hetero-chiral stereoisomers				
HET-1	0.7	0.9	0.9	-0.3
HET-2	2.5	0.9	2.0	0.8

3 Cartesian coordinates of molecular geometries.

3.1 Cartesian coordinates of the homo-chiral conformers.

Table S2: Cartesian coordinates for the equilibrium structure of HO-1.

Atoms	X	Y	Z
C	0.991080	-1.912665	-1.771172
C	1.138261	-0.541663	-1.436879
C	1.774644	-0.448078	-0.158621
C	2.016432	-1.788899	0.282633
C	1.538135	-2.684849	-0.708951
Fe	0.000507	-1.483279	-0.000007
C	-0.989548	-1.910598	1.771735
C	-1.537208	-2.683873	0.710637
C	-2.015801	-1.788944	-0.281732
C	-1.773757	-0.447676	0.158044
C	-1.136636	-0.539928	1.436038

Atoms	X	Y	Z
H	-2.458307	-2.062774	-1.224117
P	2.143749	1.025273	0.832259
C	3.740814	1.675586	0.061159
C	4.865803	0.701940	0.431001
C	3.999037	3.042143	0.707820
C	3.659840	1.825465	-1.458629
P	-2.145075	1.024519	-0.833791
H	-0.790000	0.294107	2.020881
H	-0.516566	-2.296954	2.658120
H	-1.555794	-3.758677	0.655630
H	2.458680	-2.061814	1.225400
H	1.556464	-3.759585	-0.652740
H	0.518207	-2.299843	-2.657268
H	0.792031	0.291881	-2.022676
H	4.601918	2.222042	-1.846839
H	3.475089	0.869583	-1.945139
H	2.863798	2.512016	-1.747833
H	4.939533	3.458236	0.339837
H	3.206437	3.752651	0.468373
H	4.067075	2.964514	1.793173
H	5.821667	1.076252	0.057974
H	4.950411	0.582140	1.511563
H	4.698630	-0.281708	-0.007710
H	1.260326	1.904984	0.157742
C	-3.741800	1.674227	-0.061109
H	-1.261648	1.905698	-0.161272

Atoms	X	Y	Z
C	-4.003700	3.038699	-0.710748
C	-4.866178	0.698048	-0.425846
C	-3.657839	1.828294	1.458102
H	-5.821812	1.072541	-0.052418
H	-4.952482	0.574651	-1.505883
H	-4.697210	-0.283944	0.015843
H	-4.943653	3.454541	-0.341087
H	-3.211449	3.751064	-0.475680
H	-4.074863	2.957979	-1.795678
H	-4.599605	2.224712	1.847264
H	-3.470962	0.873954	1.946775
H	-2.862097	2.516654	1.743779

Table S3: Cartesian coordinates for the equilibrium structure of HO-2.

Atoms	X	Y	Z
C	0.377677	-2.002452	0.240393
C	1.177074	-1.187801	-0.603050
C	1.995607	-0.346432	0.216151
C	1.682207	-0.662947	1.576905
C	0.692145	-1.680213	1.589260
Fe	0.000210	0.005200	0.597465
C	-0.377634	2.007271	0.210978
C	-0.694251	1.704428	1.563816
C	-1.683634	0.686471	1.564629

Atoms	X	Y	Z
C	-1.994723	0.350310	0.208091
C	-1.175231	1.180100	-0.621898
H	-2.111835	0.217326	2.433810
P	3.154966	0.958225	-0.274958
C	4.722180	-0.001019	-0.714773
C	5.317257	-0.540912	0.591057
C	5.681760	1.016483	-1.344147
C	4.472230	-1.153493	-1.688424
P	-3.152715	-0.961936	-0.265748
H	-1.152186	1.153389	-1.697305
H	0.368131	2.710218	-0.118592
H	-0.236722	2.145155	2.432493
H	2.109284	-0.181605	2.439950
H	0.232785	-2.107995	2.463429
H	-0.368051	-2.709577	-0.080225
H	1.155612	-1.176387	-1.678761
H	5.413017	-1.661159	-1.917918
H	3.789850	-1.890183	-1.268651
H	4.048844	-0.794991	-2.627086
H	6.633534	0.535744	-1.580986
H	5.277636	1.425563	-2.271359
H	5.883457	1.847318	-0.667663
H	6.263729	-1.047102	0.388951
H	5.510945	0.261995	1.303040
H	4.648557	-1.260147	1.063728
H	2.675465	1.116460	-1.600441

Atoms	X	Y	Z
C	-4.722837	-0.010994	-0.713445
H	-2.675853	-1.134910	-1.590349
C	-5.676951	-1.035315	-1.340053
C	-5.322925	0.532508	0.588571
C	-4.475338	1.137927	-1.691903
H	-6.270720	1.034432	0.382041
H	-5.515429	-0.268025	1.303530
H	-4.657882	1.256149	1.059677
H	-6.630371	-0.559751	-1.580705
H	-5.269391	-1.446676	-2.264745
H	-5.876350	-1.864059	-0.660324
H	-5.417699	1.640372	-1.926370
H	-3.797295	1.879489	-1.273695
H	-4.047796	0.776855	-2.627686

Table S4: Cartesian coordinates for the equilibrium structure of HO-3.

Atoms	X	Y	Z
C	0.667444	-1.715875	-1.431496
C	1.675328	-0.715757	-1.438218
C	1.991061	-0.377264	-0.084067
C	1.158654	-1.189037	0.751174
C	0.350676	-2.010952	-0.076939
Fe	0.000550	-0.004255	-0.468171
C	-0.663159	1.697816	-1.450526

Atoms	X	Y	Z
C	-0.351328	2.006078	-0.097826
C	-1.161962	1.191990	0.735584
C	-1.991477	0.372366	-0.094753
C	-1.671002	0.697601	-1.450918
H	-1.137381	1.164602	1.811285
P	3.130582	0.883229	0.549748
C	4.811321	0.024223	0.464882
C	4.831830	-1.063362	1.545046
C	5.856085	1.099578	0.787364
C	5.104806	-0.594093	-0.902470
P	-3.135088	-0.881879	0.544174
H	-2.106733	0.235757	-2.319615
H	-0.194048	2.125302	-2.319989
H	0.399144	2.700787	0.237800
H	1.130545	-1.151323	1.826468
H	-0.401289	-2.702026	0.262829
H	0.201098	-2.151535	-2.298406
H	2.113403	-0.262116	-2.310029
H	6.092874	-1.062265	-0.898159
H	4.374808	-1.359186	-1.159928
H	5.096701	0.161005	-1.688997
H	6.854849	0.657673	0.802860
H	5.856536	1.892930	0.038394
H	5.674077	1.554215	1.761379
H	5.813323	-1.541590	1.574455
H	4.630591	-0.648212	2.533061

Atoms	X	Y	Z
H	4.090075	-1.836113	1.344002
H	3.254520	1.617266	-0.657486
C	-4.811159	-0.013033	0.467974
H	-3.268751	-1.613191	-0.663724
C	-5.862137	-1.087280	0.773456
C	-4.827825	1.059276	1.563428
C	-5.098587	0.626413	-0.890872
H	-5.806736	1.542425	1.598003
H	-4.630573	0.628948	2.545725
H	-4.081677	1.830901	1.374586
H	-6.858461	-0.640144	0.794756
H	-5.866163	-1.869243	0.012650
H	-5.683794	-1.557520	1.740766
H	-6.083398	1.101308	-0.880987
H	-4.362928	1.389894	-1.136663
H	-5.094720	-0.117324	-1.688176

Table S5: Cartesian coordinates for the equilibrium structure of HO-4.

Atoms	X	Y	Z
C	1.365211	2.496192	-0.995519
C	2.017724	1.721035	-0.001466
C	1.804121	0.335550	-0.288749
C	1.010292	0.276513	-1.477950
C	0.744532	1.602037	-1.911519

Atoms	X	Y	Z
Fe	0.000377	1.292591	-0.000406
C	-1.359509	2.491960	1.006322
C	-0.737792	1.590309	1.914236
C	-1.007379	0.268333	1.472320
C	-1.804942	0.337154	0.286096
C	-2.016810	1.724880	0.009093
H	-0.640401	-0.639188	1.920394
P	2.314241	-1.122303	0.663325
C	4.095125	-1.398443	0.094576
C	4.045658	-1.918809	-1.346597
C	4.673944	-2.477074	1.018363
C	4.954923	-0.136047	0.163747
P	-2.321143	-1.112310	-0.675498
H	-2.562138	2.113223	-0.833321
H	-1.313914	3.566557	1.051653
H	-0.139692	1.862415	2.766629
H	0.643122	-0.627249	-1.933404
H	0.149906	1.881228	-2.764056
H	1.321712	3.571164	-1.033464
H	2.560477	2.102608	0.845686
H	5.974493	-0.357705	-0.163271
H	4.562436	0.650238	-0.478435
H	5.007823	0.252387	1.181211
H	5.692487	-2.725101	0.711461
H	4.713667	-2.136057	2.053977
H	4.080741	-3.391174	0.985851

Atoms	X	Y	Z
H	5.053908	-2.158131	-1.691788
H	3.440224	-2.822326	-1.423818
H	3.629713	-1.172857	-2.023599
H	2.626520	-0.429206	1.861190
C	-4.096258	-1.397288	-0.093057
H	-2.645718	-0.407246	-1.863017
C	-4.684690	-2.461179	-1.027826
C	-4.031924	-1.940045	1.339274
C	-4.956758	-0.134129	-0.133569
H	-5.036393	-2.185567	1.691070
H	-3.424930	-2.844016	1.396299
H	-3.609681	-1.204293	2.023531
H	-5.700695	-2.712751	-0.715462
H	-4.733533	-2.104315	-2.057682
H	-4.092291	-3.376303	-1.014780
H	-5.973191	-0.361599	0.199206
H	-4.558457	0.641617	0.517779
H	-5.019145	0.271000	-1.143932

3.2 Cartesian coordinates of the hetero-chiral conformers.

Table S6: Cartesian coordinates for the equilibrium structure of HET-1.

Atoms	X	Y	Z
C	1.478328	2.657362	-0.828968

Atoms	X	Y	Z
C	2.014813	1.820652	0.184609
C	1.792926	0.455392	-0.184440
C	1.110648	0.472863	-1.442360
C	0.916230	1.822797	-1.834544
Fe	-0.002052	1.450912	-0.011443
C	-1.538847	2.640716	0.711969
C	-0.938348	1.917912	1.779818
C	-1.060257	0.533290	1.494314
C	-1.743075	0.382345	0.245246
C	-2.036008	1.700892	-0.228839
H	-0.666569	-0.272049	2.090418
P	2.222727	-0.961914	0.864039
C	3.792675	-1.635587	0.058339
C	3.645462	-1.873554	-1.445230
C	4.098031	-2.960572	0.768184
C	4.920701	-0.631216	0.320195
P	-2.039873	-1.219342	-0.552176
H	-2.526905	1.931916	-1.158095
H	-1.581961	3.712436	0.619579
H	-0.444621	2.344950	2.635442
H	0.771682	-0.398645	-1.974643
H	0.405149	2.157288	-2.721008
H	1.469036	3.733665	-0.822533
H	2.480861	2.148106	1.098085
H	5.863606	-1.016716	-0.074064
H	4.721787	0.324191	-0.165397

Atoms	X	Y	Z
H	5.052256	-0.448648	1.387213
H	5.025588	-3.387592	0.380255
H	4.215392	-2.820408	1.843007
H	3.303894	-3.691070	0.607111
H	4.573139	-2.285069	-1.852394
H	2.843772	-2.581069	-1.657901
H	3.430778	-0.948358	-1.976775
H	1.316928	-1.880588	0.278808
C	-3.839966	-1.589247	-0.115050
H	-2.281865	-0.712866	-1.854764
C	-4.233176	-2.823086	-0.936776
C	-3.895123	-1.919647	1.381067
C	-4.786972	-0.430194	-0.428312
H	-4.910608	-2.206528	1.662833
H	-3.230307	-2.746820	1.631720
H	-3.610845	-1.060141	1.987840
H	-5.255239	-3.123240	-0.695214
H	-4.192361	-2.618913	-2.007788
H	-3.576340	-3.667959	-0.728420
H	-5.813406	-0.703542	-0.169008
H	-4.526556	0.462354	0.137587
H	-4.766737	-0.177073	-1.488704

Table S7: Cartesian coordinates for the equilibrium structure of HET-2.

Atoms	X	Y	Z
C	0.365576	-2.037092	-0.219583
C	1.107641	-1.044076	-0.910964
C	1.973136	-0.388522	0.022436
C	1.744796	-0.997145	1.297210
C	0.756676	-2.005511	1.147048
Fe	0.002271	-0.159024	0.581837
C	-0.378488	1.873061	0.749713
C	-0.693211	1.218388	1.972168
C	-1.682318	0.236714	1.701220
C	-1.994723	0.277317	0.304692
C	-1.176776	1.299162	-0.273718
H	-2.108809	-0.449465	2.412656
P	3.048459	1.005528	-0.411180
C	4.744426	0.195047	-0.603935
C	5.137045	-0.673743	0.591835
C	5.749181	1.341883	-0.769248
C	4.710317	-0.656153	-1.878551
P	-3.154689	-0.859026	-0.502729
H	-1.152065	1.561033	-1.316910
H	0.365845	2.638822	0.613683
H	-0.235790	1.409483	2.927518
H	2.225972	-0.711503	2.216332
H	0.348654	-2.616338	1.933866
H	-0.394639	-2.670638	-0.642952

Atoms	X	Y	Z
H	1.016822	-0.795771	-1.954434
H	5.695262	-1.090285	-2.063762
H	3.996400	-1.474808	-1.789632
H	4.436823	-0.059642	-2.749454
H	6.751343	0.940238	-0.934850
H	5.496135	1.975633	-1.619548
H	5.787416	1.970486	0.121685
H	6.128903	-1.105467	0.432660
H	5.171188	-0.089941	1.512099
H	4.436179	-1.493718	0.737049
H	3.239862	1.486727	0.909198
C	-4.721281	0.181966	-0.681323
H	-2.676150	-0.672943	-1.824727
C	-5.681636	-0.639671	-1.550170
C	-5.316060	0.369869	0.719163
C	-4.470475	1.545006	-1.327757
H	-6.262230	0.911496	0.653399
H	-5.510355	-0.588420	1.201904
H	-4.647173	0.943778	1.360337
H	-6.633234	-0.113896	-1.655514
H	-5.277931	-0.797444	-2.551409
H	-5.883707	-1.616155	-1.109382
H	-5.410892	2.095177	-1.419632
H	-3.787480	2.149572	-0.733957
H	-4.047317	1.438406	-2.326933

4 Measured rotational transitions.

Table S8: Assigned rotational transitions for the homo-chiral isomer HO-1.

J'	K_a'	K_c'	J"	K_a"	K_c"	ν_{obs}/MHz	ν_{calc}/MHz	$\Delta\nu$/kHz
7	1	7	6	0	6	2079.702	2079.707	-5.5
15	6	9	15	5	10	2168.336	2168.327	9.8
14	6	8	14	5	9	2235.110	2235.111	-1.4
6	2	5	5	1	4	2282.084	2282.076	7.1
13	6	7	13	5	8	2282.591	2282.584	6.9
8	1	7	7	2	6	2292.075	2292.075	-0.4
8	0	8	7	1	7	2315.248	2315.252	-4.3
12	6	7	12	5	8	2333.275	2333.284	-8.6
4	3	2	3	2	1	2334.313	2334.312	1.1
11	6	5	11	5	6	2337.645	2337.644	1.1
8	1	8	7	0	7	2347.376	2347.374	2.2
10	6	5	10	5	6	2355.907	2355.916	-8.8
4	3	1	3	2	2	2356.880	2356.888	-8.2
9	6	3	9	5	4	2363.343	2363.341	1.7
9	6	4	9	5	5	2364.380	2364.380	-0.0
8	6	2	8	5	3	2370.503	2370.497	6.5
8	6	3	8	5	4	2370.800	2370.800	-0.3
7	2	6	6	1	5	2503.009	2503.010	-1.7
9	0	9	8	1	8	2602.399	2602.398	1.0
9	1	9	8	0	8	2619.950	2619.951	-1.1

J'	K_a'	K_c'	J"	K_a"	K_c"	ν_{obs}/MHz	ν_{calc}/MHz	$\Delta\nu$/kHz
5	3	3	4	2	2	2625.256	2625.252	4.1
9	1	8	8	2	7	2645.317	2645.316	0.6
19	7	13	19	6	14	2674.287	2674.286	1.7
10	2	8	9	3	7	2681.911	2681.914	-2.8
5	3	2	4	2	3	2692.612	2692.612	0.1
16	7	9	16	6	10	2694.185	2694.190	-4.6
8	2	7	7	1	6	2720.328	2720.328	-0.3
14	7	7	14	6	8	2750.911	2750.905	5.9
13	7	6	13	6	7	2768.617	2768.621	-4.7
13	7	7	13	6	8	2771.214	2771.213	1.5
4	4	1	3	3	0	2779.511	2779.507	3.9
4	4	0	3	3	1	2779.841	2779.839	1.8
10	7	3	10	6	4	2799.256	2799.255	0.3
10	7	4	10	6	5	2799.362	2799.357	5.6
8	7	1	8	6	2	2808.698	2808.698	0.5
8	7	2	8	6	3	2808.698	2808.703	-4.8
10	0	10	9	1	9	2886.378	2886.377	1.1
6	3	4	5	2	3	2894.922	2894.920	2.3
10	1	10	9	0	9	2895.724	2895.723	0.7
9	2	8	8	1	7	2941.412	2941.409	3.3
10	1	9	9	2	8	2979.401	2979.404	-3.9
5	4	2	4	3	1	3094.217	3094.216	0.6
5	4	1	4	3	2	3096.571	3096.566	4.8
11	2	9	10	3	8	3102.963	3102.957	6.4
7	3	5	6	2	4	3140.763	3140.759	4.7
11	0	11	10	1	10	3168.524	3168.524	0.6

J'	K_a'	K_c'	J"	K_a"	K_c"	ν_{obs}/MHz	ν_{calc}/MHz	$\Delta\nu$/kHz
10	2	9	9	1	8	3172.302	3172.301	0.8
11	1	11	10	0	10	3173.402	3173.401	1.1
14	8	6	14	7	7	3210.047	3210.047	0.1
14	8	7	14	7	8	3210.364	3210.359	5.7
13	8	5	13	7	6	3219.912	3219.925	-12.1
13	8	6	13	7	7	3220.038	3220.031	7.3
9	8	1	9	7	2	3241.841	3241.839	2.5
9	8	2	9	7	3	3241.841	3241.839	2.2
11	1	10	10	2	9	3296.287	3296.287	0.1
13	3	10	12	4	9	3372.062	3372.058	4.0
6	4	2	5	3	3	3414.677	3414.677	-0.3
11	2	10	10	1	9	3415.669	3415.668	0.7
12	0	12	11	1	11	3449.656	3449.653	3.4
12	2	10	11	3	9	3506.117	3506.116	1.5
9	3	7	8	2	6	3568.381	3568.382	-1.4
12	1	11	11	2	10	3599.867	3599.860	6.2
13	9	4	13	8	5	3662.488	3662.485	3.0
13	9	5	13	8	6	3662.488	3662.488	0.2
12	9	3	12	8	4	3667.679	3667.678	0.7
12	9	4	12	8	5	3667.679	3667.679	0.0
12	2	11	11	1	10	3670.737	3670.735	2.3
11	9	2	11	8	3	3671.739	3671.742	-2.8
11	9	3	11	8	4	3671.739	3671.742	-2.9
10	9	1	10	8	2	3674.863	3674.859	4.0
10	9	2	10	8	3	3674.863	3674.859	4.0
7	4	4	6	3	3	3708.365	3708.357	8.3

J'	K_a'	K_c'	J"	K_a"	K_c"	ν_{obs}/MHz	ν_{calc}/MHz	$\Delta\nu$/kHz
13	0	13	12	1	12	3730.222	3730.234	-12.7
13	1	13	12	0	12	3731.498	3731.504	-5.9
7	4	3	6	3	4	3736.424	3736.419	4.9
10	3	8	9	2	7	3759.101	3759.098	3.4
14	3	11	13	4	10	3841.047	3841.041	6.8
6	5	2	5	4	1	3844.591	3844.598	-6.8
6	5	1	5	4	2	3844.781	3844.785	-4.5
8	3	5	7	2	6	3861.631	3861.630	1.0
13	2	11	12	3	10	3885.636	3885.637	-0.7
13	1	12	12	2	11	3894.178	3894.172	6.2
13	2	12	12	1	11	3934.893	3934.892	0.9
11	3	9	10	2	8	3942.798	3942.801	-3.3
8	4	5	7	3	4	3997.359	3997.358	0.9
14	0	14	13	1	13	4010.529	4010.529	0.1
14	1	14	13	0	13	4011.167	4011.165	1.4
8	4	4	7	3	5	4065.922	4065.923	-1.0
12	3	10	11	2	9	4127.396	4127.399	-3.4
7	5	3	6	4	2	4159.625	4159.625	0.1
7	5	2	6	4	3	4160.565	4160.564	1.5
14	1	13	13	2	12	4182.502	4182.501	1.2
14	2	13	13	1	12	4205.302	4205.298	4.2
14	2	12	13	3	11	4240.006	4240.013	-7.4
9	4	6	8	3	5	4265.281	4265.278	3.3
6	6	1	5	5	0	4277.759	4277.760	-1.2
6	6	0	5	5	1	4277.759	4277.762	-2.4
15	0	15	14	1	14	4290.680	4290.676	4.5

J'	K_a'	K_c'	J"	K_a"	K_c"	ν_{obs}/MHz	ν_{calc}/MHz	$\Delta\nu$/kHz
15	1	15	14	0	14	4291.000	4290.992	7.9
15	1	14	14	2	13	4467.170	4467.171	-0.8
8	5	4	7	4	3	4472.717	4472.717	0.1
8	5	3	7	4	4	4476.158	4476.156	1.6
15	2	14	14	1	13	4479.669	4479.670	-0.8
16	0	16	15	1	15	4570.752	4570.749	3.3
16	1	16	15	0	15	4570.904	4570.905	-1.1
15	2	13	14	3	12	4571.781	4571.791	-9.6
7	6	2	6	5	1	4593.818	4593.814	4.3
7	6	1	6	5	2	4593.818	4593.827	-8.6
16	1	15	15	2	14	4749.693	4749.695	-2.7
15	3	13	14	2	12	4755.938	4755.936	2.5
10	4	6	9	3	7	4776.892	4776.891	1.4
9	5	5	8	4	4	4781.927	4781.928	-1.5
9	5	4	8	4	5	4792.169	4792.167	1.2
17	0	17	16	1	16	4850.784	4850.786	-1.5
17	1	17	16	0	16	4850.861	4850.862	-0.8
16	2	14	15	3	13	4885.743	4885.736	6.7
8	6	3	7	5	2	4909.502	4909.511	-8.3
8	6	2	7	5	3	4909.586	4909.588	-2.5
16	3	14	15	2	13	4998.429	4998.427	2.3
7	7	1	6	6	0	5026.778	5026.775	3.0
7	7	0	6	6	1	5026.778	5026.775	2.9
17	1	16	16	2	15	5031.003	5031.005	-2.3
17	2	16	16	1	15	5034.592	5034.584	8.2
10	5	6	9	4	5	5083.963	5083.964	-1.0

J'	K_a'	K_c'	J"	K_a"	K_c"	ν_{obs}/MHz	ν_{calc}/MHz	$\Delta\nu$/kHz
10	5	5	9	4	6	5110.140	5110.138	1.4
18	1	18	17	0	17	5130.829	5130.843	-13.3
11	4	7	10	3	8	5178.669	5178.671	-2.3
17	2	15	16	3	14	5186.902	5186.908	-6.1
9	6	4	8	5	3	5224.494	5224.502	-8.5
9	6	3	8	5	4	5224.836	5224.837	-0.8
17	3	15	16	2	14	5253.772	5253.776	-3.6
18	1	17	17	2	16	5311.653	5311.650	3.3
18	2	17	17	1	16	5313.529	5313.527	2.1
8	7	2	7	6	1	5342.886	5342.884	2.0
8	7	1	7	6	2	5342.886	5342.885	1.2
11	5	7	10	4	6	5373.782	5373.787	-4.9
19	0	19	18	1	18	5410.806	5410.817	-10.1
11	5	6	10	4	7	5433.061	5433.056	5.3
18	2	16	17	3	15	5479.558	5479.555	2.7
18	3	15	17	4	14	5504.019	5504.019	-0.5
18	3	16	17	2	15	5518.249	5518.253	-3.8
10	6	5	9	5	4	5538.243	5538.242	1.4
10	6	4	9	5	5	5539.406	5539.410	-4.2
19	1	18	18	2	17	5591.948	5591.939	8.4
19	2	18	18	1	17	5592.910	5592.914	-4.5
12	4	8	11	3	9	5626.579	5626.584	-5.0
9	7	3	8	6	2	5658.775	5658.773	1.8
9	7	2	8	6	3	5658.775	5658.779	-3.8
20	0	20	19	1	19	5690.837	5690.824	13.7
20	1	20	19	0	19	5690.837	5690.832	5.0

J'	K_a'	K_c'	J"	K_a"	K_c"	ν_{obs}/MHz	ν_{calc}/MHz	$\Delta\nu$/kHz
12	5	7	11	4	8	5765.904	5765.893	11.7
8	8	0	7	7	1	5775.775	5775.775	-0.0
8	8	1	7	7	0	5775.775	5775.775	-0.0
11	6	6	10	5	5	5849.829	5849.829	0.2
11	6	5	10	5	6	5853.302	5853.305	-2.6
21	0	21	20	1	20	5970.827	5970.829	-2.4
21	1	21	20	0	20	5970.827	5970.833	-6.6
9	8	2	8	7	1	6091.912	6091.915	-3.1
9	8	1	8	7	2	6091.912	6091.915	-3.1
12	6	7	11	5	6	6157.742	6157.747	-4.9
12	6	6	11	5	7	6166.879	6166.883	-4.4
22	0	22	21	1	21	6250.840	6250.833	6.8
22	1	22	21	0	21	6250.840	6250.835	4.8
11	7	5	10	6	4	6289.093	6289.094	-0.5
11	7	4	10	6	5	6289.206	6289.206	-0.6
10	8	3	9	7	2	6407.899	6407.899	-0.5
10	8	2	9	7	3	6407.899	6407.900	-0.9
22	1	21	21	2	20	6432.037	6432.041	-3.7
22	2	21	21	1	20	6432.159	6432.170	-11.3
16	5	12	15	4	11	6441.743	6441.739	3.8
13	6	8	12	5	7	6459.481	6459.484	-2.8
9	9	1	8	8	0	6524.761	6524.759	2.4
9	9	0	8	8	1	6524.761	6524.759	2.4
23	0	23	22	1	22	6530.837	6530.836	1.0
23	1	23	22	0	22	6530.837	6530.837	0.1
12	7	6	11	6	5	6602.956	6602.959	-2.6

J'	K_a'	K_c'	J"	K_a"	K_c"	ν_{obs}/MHz	ν_{calc}/MHz	$\Delta\nu$/kHz
12	7	5	11	6	6	6603.339	6603.339	0.4
23	1	22	22	2	21	6712.000	6712.000	0.4
23	2	22	22	1	21	6712.075	6712.065	10.3
11	8	4	10	7	3	6723.610	6723.604	6.5
11	8	3	10	7	4	6723.610	6723.606	4.4
10	9	1	9	8	2	6840.919	6840.919	0.0
10	9	2	9	8	1	6840.919	6840.919	0.0
15	5	10	14	4	11	6909.874	6909.872	2.3
13	7	7	12	6	6	6915.378	6915.380	-1.7
13	7	6	12	6	7	6916.506	6916.509	-2.4
15	6	10	14	5	9	7026.866	7026.873	-7.0
12	8	5	11	7	4	7038.883	7038.879	4.6
12	8	4	11	7	5	7038.883	7038.888	-4.9
11	9	3	10	8	2	7156.968	7156.964	3.8
11	9	2	10	8	3	7156.968	7156.964	3.8
14	7	8	13	6	7	7225.627	7225.629	-2.1
14	7	7	13	6	8	7228.655	7228.652	2.8
10	10	0	9	9	1	7273.723	7273.725	-2.1
12	9	4	11	8	3	7472.798	7472.801	-3.5
12	9	3	11	8	4	7472.798	7472.802	-3.7
15	7	9	14	6	8	7532.538	7532.538	0.3
15	7	8	14	6	9	7539.945	7539.948	-3.2
11	10	1	10	9	2	7589.905	7589.902	3.3
11	10	2	10	9	1	7589.905	7589.902	3.3
14	8	7	13	7	6	7667.371	7667.366	4.5
14	8	6	13	7	7	7667.503	7667.486	16.7

J'	K_a'	K_c'	J"	K_a"	K_c"	ν_{obs}/MHz	ν_{calc}/MHz	$\Delta\nu$/kHz
13	9	5	12	8	4	7788.328	7788.325	2.8
13	9	4	12	8	5	7788.328	7788.325	2.1
12	10	3	11	9	2	7905.979	7905.988	-8.8
12	10	2	11	9	3	7905.979	7905.988	-8.8
15	8	8	14	7	7	7980.063	7980.063	0.4
15	8	7	14	7	8	7980.410	7980.419	-8.0

Table S9: Assigned rotational transitions for the singly substituted ^{56}Fe isotopologue of the hetero-chiral isomer HET-1.

J'	K_a'	K_c'	J"	K_a"	K_c"	ν_{obs}/MHz	ν_{calc}/MHz	$\Delta\nu$/kHz
3	3	1	2	2	0	2070.806	2070.796	10.2
3	3	0	2	2	1	2074.494	2074.495	-1.0
5	2	4	4	1	3	2075.438	2075.442	-3.8
7	1	7	6	0	6	2075.787	2075.788	-1.3
16	5	12	16	4	13	2104.690	2104.687	3.0
7	2	6	6	2	5	2150.364	2150.368	-3.8
17	5	13	17	4	14	2166.517	2166.521	-3.6
17	6	11	17	5	12	2179.662	2179.658	3.8
5	2	3	4	1	3	2184.873	2184.863	9.6
7	3	5	6	3	4	2193.148	2193.146	2.5
7	4	4	6	4	3	2193.507	2193.515	-8.6
7	4	3	6	4	2	2195.024	2195.023	1.0
7	3	4	6	3	3	2219.501	2219.498	3.0
8	1	7	7	2	6	2225.759	2225.754	4.8

J'	K_a'	K_c'	J"	K_a"	K_c"	ν_{obs}/MHz	ν_{calc}/MHz	$\Delta\nu$/kHz
7	1	6	6	1	5	2231.129	2231.127	2.2
17	4	14	17	3	15	2231.908	2231.918	-10.4
16	6	10	16	5	11	2269.171	2269.174	-3.0
14	1	13	14	0	14	2275.011	2275.023	-12.8
7	2	5	6	2	4	2277.521	2277.519	2.1
14	2	13	14	1	14	2288.463	2288.459	3.6
8	0	8	7	1	7	2295.788	2295.793	-5.5
8	0	8	7	0	7	2323.433	2323.434	-0.5
16	3	14	16	2	15	2334.826	2334.824	2.5
15	6	9	15	5	10	2337.905	2337.908	-4.0
8	1	8	7	0	7	2339.461	2339.457	4.0
4	3	2	3	2	1	2374.454	2374.450	3.7
4	3	1	3	2	1	2376.067	2376.067	-0.4
18	4	15	18	3	16	2382.355	2382.351	4.0
14	6	8	14	5	9	2388.027	2388.015	11.9
4	3	2	3	2	2	2391.519	2391.529	-9.4
4	3	1	3	2	2	2393.145	2393.146	-1.0
13	6	8	13	5	8	2421.986	2421.986	0.8
13	6	7	13	5	8	2423.441	2423.428	12.6
18	6	13	18	5	14	2424.600	2424.604	-3.6
14	6	9	14	5	10	2434.400	2434.402	-2.1
8	2	7	7	2	6	2446.927	2446.924	2.9
12	6	6	12	5	7	2448.122	2448.124	-1.8
12	6	7	12	5	8	2459.056	2459.064	-8.2
11	6	5	11	5	6	2465.320	2465.320	-0.2
15	2	14	15	1	15	2466.125	2466.127	-2.1

J'	K_a'	K_c'	J"	K_a"	K_c"	ν_{obs}/MHz	ν_{calc}/MHz	$\Delta\nu$/kHz
17	2	15	17	1	16	2467.351	2467.351	-0.7
11	6	6	11	5	7	2470.007	2470.004	2.9
11	6	5	11	5	7	2470.182	2470.187	-4.9
20	6	15	20	5	16	2484.122	2484.121	1.8
9	6	3	9	5	4	2485.748	2485.738	10.4
9	6	4	9	5	5	2486.355	2486.356	-1.4
8	6	2	8	5	3	2491.575	2491.578	-2.8
8	6	3	8	5	4	2491.755	2491.758	-2.9
7	6	2	7	5	2	2495.566	2495.560	6.5
7	6	1	7	5	2	2495.566	2495.560	6.1
7	6	2	7	5	3	2495.605	2495.602	3.1
7	6	1	7	5	3	2495.605	2495.602	2.7
8	6	3	7	6	2	2500.970	2500.969	1.4
8	6	2	7	6	1	2500.970	2500.971	-0.9
8	3	6	7	3	5	2505.845	2505.851	-6.3
6	2	4	5	1	4	2509.428	2509.428	-0.3
8	1	7	7	1	6	2523.280	2523.283	-3.6
7	2	6	6	1	5	2528.654	2528.656	-2.2
10	2	8	9	3	7	2548.568	2548.566	1.9
8	3	5	7	3	4	2554.345	2554.341	3.9
9	0	9	8	1	8	2583.433	2583.431	1.6
9	1	8	8	2	7	2584.787	2584.782	5.1
9	1	9	8	1	8	2592.456	2592.455	0.9
9	0	9	8	0	8	2599.457	2599.455	1.6
8	2	6	7	2	5	2607.207	2607.207	0.1
9	1	9	8	0	8	2608.479	2608.479	0.0

J'	K_a'	K_c'	J"	K_a"	K_c"	ν_{obs}/MHz	ν_{calc}/MHz	$\Delta\nu$/kHz
5	3	3	4	2	2	2665.287	2665.286	1.0
5	3	2	4	2	2	2671.665	2671.673	-8.3
12	3	9	11	4	8	2694.415	2694.410	4.3
20	4	17	20	3	18	2711.912	2711.910	1.4
5	3	3	4	2	3	2714.893	2714.878	15.1
5	3	2	4	2	3	2721.259	2721.265	-5.7
19	7	12	19	6	13	2721.740	2721.739	1.1
8	2	7	7	1	6	2744.450	2744.453	-2.8
9	1	8	8	1	7	2805.956	2805.951	4.7
9	6	4	8	6	3	2816.149	2816.131	18.3
9	6	3	8	6	2	2816.149	2816.142	7.3
9	3	7	8	3	6	2816.367	2816.377	-10.8
9	5	5	8	5	4	2821.533	2821.533	0.3
9	5	4	8	5	3	2821.988	2821.982	5.7
23	7	17	23	6	18	2822.150	2822.142	7.6
17	2	16	17	1	17	2823.416	2823.417	-0.5
19	7	13	19	6	14	2823.751	2823.760	-9.0
17	7	10	17	6	11	2827.516	2827.519	-2.9
9	4	6	8	4	5	2828.245	2828.245	0.1
9	4	5	8	4	4	2837.637	2837.640	-3.0
4	4	1	3	3	0	2839.282	2839.285	-2.7
4	4	0	3	3	1	2839.525	2839.531	-5.5
7	2	5	6	1	5	2857.875	2857.874	1.5
17	7	11	17	6	12	2858.721	2858.727	-6.2
16	7	9	16	6	10	2860.679	2860.685	-5.3
10	0	10	9	1	9	2867.257	2867.260	-3.1

J'	K_a'	K_c'	J"	K_a"	K_c"	ν_{obs}/MHz	ν_{calc}/MHz	$\Delta\nu$/kHz
10	1	10	9	1	9	2872.230	2872.230	-0.4
10	0	10	9	0	9	2876.294	2876.284	9.9
10	1	10	9	0	9	2881.249	2881.254	-4.8
15	7	9	15	6	9	2884.886	2884.890	-4.5
15	7	8	15	6	9	2885.299	2885.303	-3.9
21	4	18	21	3	19	2886.300	2886.311	-10.9
15	7	9	15	6	10	2892.853	2892.855	-1.4
9	3	6	8	3	5	2895.030	2895.033	-3.0
18	6	12	17	7	11	2902.108	2902.101	6.6
25	7	19	25	6	20	2902.550	2902.553	-3.4
14	7	8	14	6	8	2903.620	2903.625	-5.1
14	7	7	14	6	8	2903.783	2903.778	5.6
14	7	8	14	6	9	2907.144	2907.145	-1.2
14	7	7	14	6	9	2907.298	2907.297	0.7
13	7	7	13	6	7	2917.721	2917.731	-9.7
13	7	6	13	6	7	2917.787	2917.783	5.0
13	7	7	13	6	8	2919.175	2919.173	1.5
10	1	9	9	2	8	2926.488	2926.484	4.1
12	7	5	12	6	6	2928.459	2928.454	4.6
12	7	6	12	6	7	2928.973	2928.980	-7.8
9	2	7	8	2	6	2929.847	2929.854	-6.8
6	3	4	5	2	3	2937.382	2937.379	3.4
10	7	4	10	6	4	2942.700	2942.694	5.6
10	7	3	10	6	4	2942.700	2942.695	4.7
10	7	4	10	6	5	2942.750	2942.749	1.1
12	2	10	11	3	8	2944.862	2944.858	3.7

J'	K_a'	K_c'	J"	K_a"	K_c"	ν_{obs}/MHz	ν_{calc}/MHz	$\Delta\nu$/kHz
9	7	2	9	6	3	2947.228	2947.232	-4.8
9	7	3	9	6	4	2947.228	2947.246	-18.4
8	7	1	8	6	2	2950.517	2950.511	5.4
8	7	2	8	6	3	2950.517	2950.514	2.7
7	7	0	7	6	1	2952.800	2952.801	-0.7
7	7	1	7	6	2	2952.800	2952.801	-1.1
6	3	3	5	2	3	2956.098	2956.106	-8.6
9	2	8	8	1	7	2961.140	2961.149	-9.3
11	2	9	10	3	8	2971.887	2971.887	0.9
6	3	4	5	2	4	3046.807	3046.800	7.0
6	3	3	5	2	4	3065.535	3065.527	7.2
10	1	9	9	1	8	3081.679	3081.682	-2.7
10	3	8	9	3	7	3123.992	3123.984	8.9
10	8	2	9	8	1	3124.830	3124.836	-5.3
10	7	4	9	7	3	3127.700	3127.700	-0.5
10	7	3	9	7	2	3127.700	3127.701	-1.2
10	5	6	9	5	5	3139.407	3139.409	-1.7
10	5	5	9	5	4	3140.645	3140.642	2.9
10	4	7	9	4	6	3146.145	3146.146	-1.3
11	0	11	10	1	10	3148.755	3148.758	-2.9
5	4	2	4	3	1	3150.287	3150.286	1.1
5	4	1	4	3	1	3150.401	3150.402	-1.3
11	1	11	10	1	10	3151.450	3151.447	2.3
5	4	2	4	3	2	3151.910	3151.904	5.6
5	4	1	4	3	2	3152.018	3152.020	-1.8
11	0	11	10	0	10	3153.723	3153.727	-4.0

J'	K_a'	K_c'	J"	K_a"	K_c"	ν_{obs}/MHz	ν_{calc}/MHz	$\Delta\nu$/kHz
11	1	11	10	0	10	3156.417	3156.417	-0.1
10	4	6	9	4	5	3165.486	3165.487	-1.3
10	2	9	9	1	8	3184.891	3184.893	-2.4
7	3	5	6	2	4	3187.506	3187.511	-5.2
7	2	6	6	1	6	3192.716	3192.710	6.2
15	4	11	14	5	10	3209.575	3209.581	-6.1
7	3	4	6	2	4	3232.591	3232.590	0.8
8	2	6	7	1	6	3233.945	3233.954	-8.7
10	3	7	9	3	6	3238.422	3238.402	19.3
10	2	8	9	2	7	3243.724	3243.726	-1.9
11	1	10	10	2	9	3250.875	3250.874	1.3
20	8	12	20	7	13	3264.488	3264.483	4.8
19	8	12	19	7	12	3295.400	3295.403	-3.1
19	8	11	19	7	12	3296.108	3296.104	4.1
19	8	12	19	7	13	3306.468	3306.468	0.3
11	2	10	10	2	9	3316.427	3316.428	-1.6
18	8	11	18	7	12	3325.876	3325.875	1.3
18	8	10	18	7	12	3326.148	3326.167	-19.5
17	8	9	17	7	10	3340.496	3340.498	-2.2
17	8	10	17	7	11	3342.792	3342.793	-1.2
17	8	9	17	7	11	3342.910	3342.908	2.5
11	1	10	10	1	9	3354.082	3354.085	-2.4
16	8	8	16	7	9	3356.211	3356.220	-8.8
16	8	9	16	7	10	3357.218	3357.209	8.8
15	8	7	15	7	8	3368.868	3368.864	4.8
15	8	8	15	7	9	3369.259	3369.262	-2.3

J'	K_a'	K_c'	J"	K_a"	K_c"	ν_{obs}/MHz	ν_{calc}/MHz	$\Delta\nu$/kHz
14	8	6	14	7	7	3379.031	3379.024	6.8
14	8	7	14	7	8	3379.177	3379.172	4.6
12	2	10	11	3	9	3383.469	3383.465	3.9
7	3	5	6	2	5	3389.578	3389.578	0.3
12	8	5	12	7	5	3393.569	3393.565	3.9
12	8	4	12	7	5	3393.569	3393.566	3.6
11	8	3	11	7	4	3398.582	3398.574	8.4
11	8	4	11	7	5	3398.582	3398.578	4.3
10	8	2	10	7	3	3402.407	3402.407	-0.3
10	8	3	10	7	4	3402.407	3402.408	-1.2
8	8	0	8	7	1	3407.355	3407.353	2.4
8	8	1	8	7	2	3407.355	3407.353	2.4
8	3	6	7	2	5	3415.838	3415.844	-6.3
11	2	10	10	1	9	3419.638	3419.639	-1.6
11	3	9	10	3	8	3428.103	3428.100	2.8
12	0	12	11	1	11	3428.885	3428.887	-2.0
12	1	12	11	1	11	3430.328	3430.322	5.3
12	0	12	11	0	11	3431.574	3431.577	-3.2
12	1	12	11	0	11	3433.014	3433.012	2.2
7	3	4	6	2	5	3434.657	3434.657	0.4
11	8	3	10	8	2	3439.436	3439.442	-5.7
11	7	5	10	7	4	3443.273	3443.272	1.4
11	7	4	10	7	3	3443.273	3443.275	-1.9
11	6	6	10	6	5	3449.273	3449.268	4.8
11	6	5	10	6	4	3449.398	3449.396	1.2
11	5	7	10	5	6	3458.400	3458.408	-8.5

J'	K_a'	K_c'	J"	K_a"	K_c"	ν_{obs}/MHz	ν_{calc}/MHz	$\Delta\nu$/kHz
6	4	3	5	3	2	3458.471	3458.470	1.1
6	4	2	5	3	2	3459.045	3459.045	-0.1
11	5	6	10	5	5	3461.412	3461.410	1.3
6	4	3	5	3	3	3464.856	3464.857	-1.4
6	4	2	5	3	3	3465.429	3465.433	-3.2
11	4	7	10	4	6	3499.445	3499.447	-2.4
8	3	5	7	2	5	3509.413	3509.413	-0.0
11	2	9	10	2	8	3547.307	3547.304	2.1
12	1	11	11	2	10	3560.553	3560.555	-1.8
11	3	8	10	3	7	3580.066	3580.064	2.4
5	5	1	4	4	0	3606.096	3606.093	3.4
5	5	0	4	4	0	3606.096	3606.093	2.8
5	5	1	4	4	1	3606.096	3606.106	-9.5
5	5	0	4	4	1	3606.096	3606.106	-10.2
9	3	7	8	2	6	3625.020	3625.014	5.8
14	3	11	13	4	10	3625.968	3625.959	8.7
12	1	11	11	1	10	3626.112	3626.109	2.7
9	2	7	8	1	7	3640.525	3640.525	0.6
12	2	11	11	1	10	3666.219	3666.225	-5.4
13	0	13	12	1	12	3708.238	3708.240	-1.6
13	1	13	12	1	12	3709.000	3708.997	3.3
13	0	13	12	0	12	3709.675	3709.675	0.0
13	1	13	12	0	12	3710.430	3710.432	-2.2
8	3	6	7	2	6	3745.059	3745.061	-2.8
12	8	4	11	8	3	3754.649	3754.661	-11.8
7	4	4	6	3	3	3760.545	3760.546	-1.1

J'	K_a'	K_c'	J"	K_a"	K_c"	ν_{obs}/MHz	ν_{calc}/MHz	$\Delta\nu$/kHz
7	4	3	6	3	3	3762.631	3762.630	0.9
12	6	7	11	6	6	3767.418	3767.430	-11.9
12	6	6	11	6	5	3767.798	3767.789	8.9
13	2	11	12	3	10	3776.508	3776.509	-0.8
12	5	8	11	5	7	3778.371	3778.370	0.9
7	4	4	6	3	4	3779.269	3779.274	-5.3
12	4	9	11	4	8	3779.551	3779.547	3.7
7	4	3	6	3	4	3781.357	3781.357	0.4
12	5	7	11	5	6	3784.980	3784.985	-4.7
9	3	6	8	2	6	3797.238	3797.239	-0.7
18	9	9	18	8	10	3807.491	3807.492	-1.1
18	9	10	18	8	11	3807.770	3807.773	-3.3
10	3	8	9	2	7	3819.142	3819.144	-1.6
17	9	8	17	8	9	3819.461	3819.463	-2.1
17	9	9	17	8	10	3819.567	3819.574	-6.6
16	9	7	16	8	8	3829.415	3829.397	17.9
16	9	8	16	8	9	3829.415	3829.438	-22.7
15	9	7	15	8	7	3837.591	3837.586	4.6
15	9	6	15	8	7	3837.591	3837.586	4.2
15	9	7	15	8	8	3837.591	3837.600	-9.6
15	9	6	15	8	8	3837.591	3837.601	-10.0
8	3	5	7	2	6	3838.633	3838.630	3.1
12	2	10	11	2	9	3839.671	3839.679	-7.7
12	4	8	11	4	7	3840.213	3840.211	1.0
13	9	5	13	8	5	3849.684	3849.677	6.7
13	9	4	13	8	6	3849.684	3849.678	5.5

J'	K_a'	K_c'	J"	K_a"	K_c"	ν_{obs}/MHz	ν_{calc}/MHz	$\Delta\nu$/kHz
12	9	4	12	8	5	3853.980	3853.978	2.3
13	1	12	12	2	11	3859.126	3859.133	-6.7
10	9	1	10	8	2	3859.918	3859.925	-6.6
10	9	2	10	8	3	3859.918	3859.925	-6.6
9	9	0	9	8	1	3861.846	3861.856	-10.0
9	9	1	9	8	2	3861.846	3861.856	-10.0
13	2	12	12	2	11	3882.963	3882.967	-3.6
9	1	8	8	0	8	3898.698	3898.696	1.6
13	1	12	12	1	11	3899.243	3899.248	-5.0
12	3	9	11	3	8	3916.073	3916.064	8.6
6	5	2	5	4	1	3917.985	3917.986	-0.7
6	5	1	5	4	2	3918.107	3918.109	-2.1
13	2	12	12	1	11	3923.088	3923.082	5.9
14	0	14	13	1	13	3987.161	3987.162	-1.1
14	1	14	13	0	13	3988.314	3988.315	-0.4
11	3	9	10	2	8	4003.521	4003.518	2.9
13	3	11	12	3	10	4024.678	4024.686	-8.2
9	2	8	8	1	8	4037.874	4037.870	3.8
8	4	5	7	3	4	4051.576	4051.578	-2.8
8	4	4	7	3	4	4057.724	4057.715	8.9
13	9	5	12	9	4	4066.257	4066.253	4.7
13	9	4	12	9	3	4066.257	4066.253	4.6
10	2	8	9	1	8	4078.294	4078.300	-5.5
13	4	10	12	4	9	4093.346	4093.349	-3.3
8	4	5	7	3	5	4096.651	4096.658	-6.5
13	5	9	12	5	8	4098.968	4098.965	3.4

J'	K_a'	K_c'	J"	K_a"	K_c"	ν_{obs}/MHz	ν_{calc}/MHz	$\Delta\nu$/kHz
8	4	4	7	3	5	4102.821	4102.794	26.8
10	3	7	9	2	7	4105.794	4105.787	7.2
9	3	7	8	2	7	4114.518	4114.515	3.1
13	2	11	12	2	10	4121.422	4121.417	5.3
14	2	12	13	3	11	4146.895	4146.893	2.0
14	1	13	13	2	12	4150.000	4150.007	-6.6
14	2	13	13	2	12	4163.841	4163.838	3.0
14	1	13	13	1	12	4173.833	4173.841	-7.8
12	3	10	11	2	9	4184.590	4184.587	3.2
13	4	9	12	4	8	4186.876	4186.880	-4.4
14	2	13	13	1	12	4187.672	4187.672	0.1
7	5	3	6	4	2	4229.230	4229.224	6.2
7	5	2	6	4	3	4229.826	4229.842	-15.7
13	3	10	12	3	9	4243.613	4243.619	-6.6
20	10	10	20	9	11	4258.314	4258.312	1.6
20	10	11	20	9	12	4258.394	4258.390	3.3
15	0	15	14	1	14	4265.848	4265.852	-4.3
15	1	15	14	1	14	4266.058	4266.057	1.1
15	0	15	14	0	14	4266.247	4266.248	-0.3
15	1	15	14	0	14	4266.448	4266.453	-4.5
18	10	9	18	9	10	4279.686	4279.701	-14.8
9	3	6	8	2	7	4286.740	4286.740	0.1
17	10	7	17	9	8	4287.956	4287.958	-2.6
17	10	8	17	9	9	4287.956	4287.962	-6.4
16	10	6	16	9	7	4294.873	4294.872	1.0
16	10	7	16	9	8	4294.873	4294.873	-0.2

J'	K_a'	K_c'	J"	K_a"	K_c"	ν_{obs}/MHz	ν_{calc}/MHz	$\Delta\nu$/kHz
14	10	5	14	9	5	4305.292	4305.296	-4.1
14	10	4	14	9	5	4305.292	4305.296	-4.1
14	10	4	14	9	6	4305.292	4305.296	-4.2
14	10	5	14	9	6	4305.292	4305.296	-4.2
13	10	4	13	9	4	4309.092	4309.096	-3.8
13	10	3	13	9	4	4309.092	4309.096	-3.8
13	10	4	13	9	5	4309.092	4309.096	-3.8
13	10	3	13	9	5	4309.092	4309.096	-3.8
12	10	2	12	9	4	4312.118	4312.125	-6.8
12	10	3	12	9	3	4312.118	4312.125	-6.8
12	10	2	12	9	3	4312.118	4312.125	-6.8
12	10	3	12	9	4	4312.118	4312.125	-6.8
11	10	2	11	9	3	4314.498	4314.497	0.6
11	10	1	11	9	2	4314.498	4314.497	0.6
9	4	6	8	3	5	4325.483	4325.482	0.8
9	4	5	8	3	5	4341.014	4341.014	-0.6
13	3	11	12	2	10	4369.586	4369.594	-7.9
6	6	1	5	5	0	4372.768	4372.764	4.3
6	6	0	5	5	0	4372.768	4372.764	4.2
6	6	1	5	5	1	4372.768	4372.764	3.6
6	6	0	5	5	1	4372.768	4372.764	3.6
14	10	5	13	10	4	4377.983	4377.982	1.1
14	10	4	13	10	3	4377.983	4377.982	1.1
10	1	9	9	0	9	4380.924	4380.923	1.6
14	9	6	13	9	5	4381.791	4381.782	9.4
14	9	5	13	9	4	4381.791	4381.782	9.4

J'	K_a'	K_c'	J"	K_a"	K_c"	ν_{obs}/MHz	ν_{calc}/MHz	$\Delta\nu$/kHz
14	8	7	13	8	6	4387.184	4387.180	3.6
14	8	6	13	8	5	4387.184	4387.183	0.5
14	2	12	13	2	11	4395.069	4395.071	-1.7
14	7	8	13	7	7	4395.200	4395.201	-1.1
14	7	7	13	7	6	4395.303	4395.302	0.6
14	4	11	13	4	10	4404.134	4404.138	-4.4
14	6	9	13	6	8	4407.225	4407.230	-4.7
14	6	8	13	6	7	4409.307	4409.307	0.5
9	4	6	8	3	6	4419.047	4419.051	-4.0
14	5	10	13	5	9	4419.668	4419.674	-6.1
9	4	5	8	3	6	4434.596	4434.583	13.1
15	1	14	14	2	13	4435.843	4435.850	-7.6
11	3	8	10	2	8	4442.123	4442.125	-1.5
15	2	14	14	2	13	4443.730	4443.725	5.5
14	5	9	13	5	8	4444.732	4444.720	12.0
15	2	14	14	1	13	4457.560	4457.556	3.2
19	3	16	18	4	14	4473.312	4473.308	3.2
10	2	9	9	1	9	4475.107	4475.110	-3.5
15	2	13	14	3	12	4494.177	4494.181	-4.0
10	3	8	9	2	8	4498.521	4498.520	1.5
16	3	13	15	4	12	4536.087	4536.090	-2.4
8	5	4	7	4	3	4539.008	4539.014	-6.0
8	5	3	7	4	3	4539.188	4539.197	-8.4
8	5	4	7	4	4	4541.105	4541.097	7.3
8	5	3	7	4	4	4541.281	4541.280	1.4
11	2	9	10	1	9	4543.919	4543.922	-3.3

J'	K_a'	K_c'	J"	K_a"	K_c"	ν_{obs}/MHz	ν_{calc}/MHz	$\Delta\nu$/kHz
16	0	16	15	1	15	4544.420	4544.418	2.1
16	1	16	15	1	15	4544.520	4544.524	-4.0
16	0	16	15	0	15	4544.625	4544.623	1.9
16	1	16	15	0	15	4544.725	4544.729	-4.0
14	3	11	13	3	10	4560.828	4560.826	2.0
14	3	12	13	2	11	4565.345	4565.342	2.9
10	4	7	9	3	6	4576.598	4576.595	3.4
10	4	6	9	3	6	4611.468	4611.469	-0.7
15	2	13	14	2	12	4664.454	4664.453	0.9
7	6	2	6	5	1	4684.792	4684.791	1.6
7	6	1	6	5	1	4684.792	4684.791	1.2
7	6	2	6	5	2	4684.792	4684.798	-5.5
7	6	1	6	5	2	4684.792	4684.798	-5.9
16	1	15	15	2	14	4718.545	4718.557	-12.0
20	11	9	20	10	10	4729.951	4729.943	8.0
20	11	10	20	10	11	4729.951	4729.946	5.1
16	2	15	15	1	14	4730.847	4730.845	2.6
15	6	9	14	6	8	4733.259	4733.255	4.1
19	11	8	19	10	9	4738.284	4738.281	2.8
19	11	9	19	10	10	4738.284	4738.282	1.8
10	4	7	9	3	7	4748.812	4748.819	-7.0
17	11	6	17	10	7	4751.388	4751.387	1.0
17	11	7	17	10	8	4751.388	4751.387	0.9
16	11	5	16	10	6	4756.428	4756.419	8.9
16	11	6	16	10	7	4756.428	4756.419	8.9
14	11	4	14	10	5	4764.015	4764.020	-4.7

J'	K_a'	K_c'	J"	K_a"	K_c"	ν_{obs}/MHz	ν_{calc}/MHz	$\Delta\nu$/kHz
14	11	3	14	10	4	4764.015	4764.020	-4.7
13	11	3	13	10	4	4766.787	4766.793	-6.3
13	11	2	13	10	3	4766.787	4766.793	-6.3
12	11	2	12	10	3	4768.999	4769.004	-5.0
12	11	1	12	10	2	4768.999	4769.004	-5.0
15	3	13	14	2	12	4776.415	4776.418	-2.6
15	5	10	14	5	9	4783.363	4783.362	1.6
10	4	6	9	3	7	4783.689	4783.693	-4.0
11	4	8	10	3	7	4801.725	4801.718	7.8
12	3	9	11	2	9	4810.895	4810.884	10.8
16	2	14	15	3	13	4821.079	4821.086	-7.1
17	0	17	16	1	16	4822.924	4822.920	4.2
17	1	17	16	0	16	4823.080	4823.079	1.3
9	5	5	8	4	4	4845.965	4845.964	0.7
9	5	4	8	4	4	4846.599	4846.596	3.0
9	5	5	8	4	5	4852.107	4852.100	6.3
9	5	4	8	4	5	4852.738	4852.732	5.2
11	1	10	10	0	10	4858.719	4858.724	-4.4
11	4	7	10	3	7	4872.524	4872.513	10.9
15	4	11	14	4	10	4884.296	4884.295	1.3
16	3	14	15	3	13	4892.113	4892.127	-13.3
16	2	14	15	2	13	4933.044	4933.051	-7.1
17	3	14	16	4	13	4962.148	4962.146	2.2
8	6	3	7	5	2	4996.553	4996.529	24.2
8	6	2	7	5	3	4996.553	4996.574	-21.1
17	1	16	16	2	15	4999.376	4999.377	-0.3

J'	K_a'	K_c'	J"	K_a"	K_c"	ν_{obs}/MHz	ν_{calc}/MHz	$\Delta\nu$/kHz
12	4	9	11	3	8	5001.199	5001.201	-1.8
17	2	16	16	2	15	5001.816	5001.817	-0.7
17	1	16	16	1	15	5003.785	5003.790	-4.8
16	3	14	15	2	13	5004.086	5004.092	-5.7
17	2	16	16	1	15	5006.227	5006.230	-2.4
12	2	10	11	1	10	5029.520	5029.516	4.1
16	7	10	15	7	9	5034.961	5034.964	-3.1
16	7	9	15	7	8	5035.586	5035.582	3.4
16	5	12	15	5	11	5058.531	5058.536	-4.8
16	6	10	15	6	9	5060.201	5060.200	0.5
11	4	8	10	3	8	5088.356	5088.361	-4.4
18	0	18	17	1	17	5101.390	5101.388	2.2
18	1	18	17	0	17	5101.465	5101.469	-4.1
16	5	11	15	5	10	5128.933	5128.935	-1.9
17	2	15	16	3	14	5131.917	5131.904	13.2
12	4	8	11	3	8	5132.671	5132.661	10.3
7	7	1	6	6	0	5139.416	5139.409	6.4
7	7	0	6	6	1	5139.416	5139.409	6.4
10	5	6	9	4	5	5147.731	5147.732	-1.4
10	5	5	9	4	5	5149.594	5149.598	-3.1
11	4	7	10	3	8	5159.159	5159.156	2.7
10	5	6	9	4	6	5163.263	5163.264	-1.2
10	5	5	9	4	6	5165.122	5165.130	-7.6
17	3	15	16	3	14	5175.669	5175.682	-12.1
13	4	10	12	3	9	5178.480	5178.486	-5.6
21	12	9	21	11	10	5188.565	5188.568	-2.9

J'	K_a'	K_c'	J"	K_a"	K_c"	ν_{obs}/MHz	ν_{calc}/MHz	$\Delta\nu$/kHz
21	12	10	21	11	11	5188.565	5188.568	-3.1
20	12	8	20	11	9	5195.827	5195.827	-0.6
20	12	9	20	11	10	5195.827	5195.827	-0.7
19	4	15	18	5	14	5198.842	5198.840	2.1
19	12	7	19	11	8	5202.064	5202.064	0.1
19	12	8	19	11	9	5202.064	5202.064	0.1
17	2	15	16	2	14	5202.930	5202.945	-15.3
18	12	7	18	11	8	5207.383	5207.384	-0.9
18	12	6	18	11	7	5207.383	5207.384	-0.9
17	12	5	17	11	6	5211.877	5211.887	-9.9
17	12	6	17	11	7	5211.877	5211.887	-9.9
13	3	10	12	2	10	5214.821	5214.825	-4.1
16	12	5	16	11	6	5215.659	5215.663	-4.5
16	12	4	16	11	5	5215.659	5215.663	-4.5
15	12	3	15	11	4	5218.814	5218.799	15.4
15	12	4	15	11	5	5218.814	5218.799	15.4
14	12	3	14	11	4	5221.373	5221.371	2.6
14	12	2	14	11	3	5221.373	5221.371	2.6
17	3	15	16	2	14	5246.718	5246.722	-3.8
18	1	17	17	2	16	5279.089	5279.095	-6.4
18	2	17	17	2	16	5280.429	5280.429	0.3
18	1	17	17	1	16	5281.534	5281.535	-1.5
18	2	17	17	1	16	5282.874	5282.869	4.8
9	6	4	8	5	3	5307.705	5307.706	-1.1
9	6	3	8	5	4	5307.907	5307.903	4.3
12	3	10	11	2	10	5308.879	5308.869	9.5

J'	K_a'	K_c'	J"	K_a"	K_c"	ν_{obs}/MHz	ν_{calc}/MHz	$\Delta\nu$/kHz
17	4	14	16	4	13	5313.168	5313.173	-4.9
17	10	8	16	10	7	5325.380	5325.379	1.0
17	10	7	16	10	6	5325.380	5325.379	0.9
12	1	11	11	0	11	5331.106	5331.106	0.3
17	9	9	16	9	8	5332.293	5332.291	2.8
17	9	8	16	9	7	5332.293	5332.293	0.1
11	3	8	10	2	9	5335.525	5335.531	-6.3
14	4	11	13	3	10	5338.997	5339.005	-8.3
17	8	10	16	8	9	5342.149	5342.155	-5.5
17	8	9	16	8	8	5342.233	5342.227	5.0
12	2	11	11	1	11	5368.546	5368.531	14.3
17	6	12	16	6	11	5374.361	5374.361	-0.0
17	5	13	16	5	12	5375.008	5375.007	1.0
19	0	19	18	1	18	5379.835	5379.839	-4.7
19	1	19	18	0	18	5379.879	5379.881	-1.5
17	6	11	16	6	10	5391.105	5391.115	-9.8
13	4	9	12	3	9	5403.470	5403.477	-6.7
18	2	16	17	3	15	5431.054	5431.048	5.9
12	4	9	11	3	9	5439.806	5439.808	-1.8
17	3	14	16	3	13	5440.406	5440.405	0.7
11	5	7	10	4	6	5440.667	5440.653	14.0
11	5	6	10	4	6	5445.519	5445.520	-1.4
8	7	2	7	6	1	5451.486	5451.483	3.5
8	7	1	7	6	1	5451.486	5451.483	3.5
8	7	2	7	6	2	5451.486	5451.483	3.1
8	7	1	7	6	2	5451.486	5451.483	3.1

J'	K_a'	K_c'	J"	K_a"	K_c"	ν_{obs}/MHz	ν_{calc}/MHz	$\Delta\nu$/kHz
11	5	7	10	4	7	5475.531	5475.527	4.1
11	5	6	10	4	7	5480.389	5480.394	-5.0
17	5	12	16	5	11	5480.622	5480.631	-9.2
15	4	12	14	3	11	5489.457	5489.458	-1.8
18	3	16	17	2	15	5501.166	5501.168	-1.9
19	1	18	18	2	17	5558.213	5558.189	24.2
19	2	18	18	1	17	5560.227	5560.244	-17.0
18	4	15	17	4	14	5607.815	5607.824	-8.5
10	6	5	9	5	4	5617.925	5617.922	3.4
10	6	4	9	5	4	5617.980	5617.976	3.7
10	6	5	9	5	5	5618.560	5618.554	6.0
10	6	4	9	5	5	5618.615	5618.608	6.6
16	4	13	15	3	12	5637.532	5637.525	7.5
14	3	11	13	2	11	5654.238	5654.234	3.6
20	0	20	19	1	19	5658.282	5658.282	-0.5
20	1	20	19	0	19	5658.312	5658.303	9.7
18	8	11	17	8	10	5662.420	5662.412	7.6
18	8	10	17	8	9	5662.581	5662.590	-9.1
19	13	7	19	12	8	5663.013	5663.009	3.5
19	13	6	19	12	7	5663.013	5663.009	3.5
16	13	4	16	12	5	5673.462	5673.461	1.5
16	13	3	16	12	4	5673.462	5673.461	1.5
15	13	3	15	12	4	5675.876	5675.870	6.2
15	13	2	15	12	3	5675.876	5675.870	6.2
15	13	2	15	12	4	5675.876	5675.870	6.2
15	13	3	15	12	3	5675.876	5675.870	6.2

J'	K_a'	K_c'	J"	K_a"	K_c"	ν_{obs}/MHz	ν_{calc}/MHz	$\Delta\nu$/kHz
12	5	8	11	4	7	5719.575	5719.577	-1.5
19	2	17	18	3	16	5722.233	5722.231	2.3
12	5	7	11	4	7	5731.069	5731.058	10.7
19	3	16	18	4	15	5732.172	5732.161	10.9
9	7	3	8	6	2	5763.376	5763.374	2.0
9	7	2	8	6	2	5763.376	5763.375	1.8
9	7	3	8	6	3	5763.376	5763.377	-0.8
9	7	2	8	6	3	5763.376	5763.377	-0.9
19	3	17	18	2	16	5764.118	5764.118	0.5
12	5	8	11	4	8	5790.372	5790.372	-0.6
17	4	14	16	3	13	5791.430	5791.433	-2.9
12	5	7	11	4	8	5801.849	5801.854	-4.4
13	4	10	12	3	10	5804.784	5804.784	0.1
18	5	13	17	5	12	5835.550	5835.545	5.4
20	1	19	19	2	18	5836.931	5836.936	-4.9
20	2	19	19	2	18	5837.320	5837.324	-3.3
20	1	19	19	1	18	5837.665	5837.658	7.0
20	2	19	19	1	18	5838.048	5838.045	2.7
8	8	1	7	7	0	5906.029	5906.035	-5.9
8	8	1	7	7	1	5906.029	5906.035	-5.9
8	8	0	7	7	0	5906.029	5906.035	-5.9
8	8	0	7	7	1	5906.029	5906.035	-5.9
11	6	6	10	5	5	5926.534	5926.547	-12.6
11	6	5	10	5	6	5928.594	5928.596	-1.9
21	0	21	20	1	20	5936.730	5936.720	10.2
21	1	21	20	0	20	5936.730	5936.731	-0.3

J'	K_a'	K_c'	J"	K_a"	K_c"	ν_{obs}/MHz	ν_{calc}/MHz	$\Delta\nu$/kHz
19	12	7	18	12	6	5947.559	5947.559	-0.6
19	12	8	18	12	7	5947.559	5947.559	-0.6
18	4	15	17	3	14	5958.849	5958.851	-2.6
19	10	10	18	10	9	5959.975	5959.981	-5.8
19	10	9	18	10	8	5959.975	5959.982	-6.5
13	5	9	12	4	8	5978.331	5978.330	1.3
19	3	16	18	3	15	5978.775	5978.765	10.1
13	5	8	12	4	8	6003.183	6003.190	-6.7
20	2	18	19	3	17	6008.237	6008.237	0.3
10	7	4	9	6	3	6074.944	6074.933	11.5
10	7	3	9	6	4	6074.944	6074.947	-3.2
20	3	17	19	4	16	6077.472	6077.468	4.5
13	5	9	12	4	9	6109.780	6109.790	-10.3
21	1	20	20	2	19	6115.487	6115.498	-10.5
21	2	20	20	2	19	6115.692	6115.704	-12.1
21	1	20	20	1	19	6115.887	6115.885	1.6
21	2	20	20	1	19	6116.090	6116.091	-1.5
15	3	12	14	2	12	6125.445	6125.447	-1.7
13	5	8	12	4	9	6134.645	6134.650	-4.7
19	4	16	18	3	15	6145.200	6145.207	-6.5
14	3	12	13	2	12	6167.088	6167.083	4.2
14	4	11	13	3	11	6184.232	6184.236	-3.9
20	4	17	19	4	16	6185.978	6185.989	-11.0
19	4	15	18	4	14	6187.094	6187.087	7.5
19	5	14	18	5	13	6189.098	6189.102	-3.4
14	5	10	13	4	9	6211.138	6211.123	14.6

J'	K_a'	K_c'	J"	K_a"	K_c"	ν_{obs}/MHz	ν_{calc}/MHz	$\Delta\nu$/kHz
22	0	22	21	1	21	6215.158	6215.156	2.4
22	1	22	21	0	21	6215.158	6215.161	-2.8
9	8	2	8	7	1	6218.144	6218.136	7.7
9	8	1	8	7	2	6218.144	6218.136	7.6
12	6	7	11	5	6	6232.564	6232.567	-3.0
12	6	6	11	5	6	6233.113	6233.109	4.3
12	6	7	11	5	7	6237.431	6237.434	-3.4
12	6	6	11	5	7	6237.962	6237.976	-14.1
14	5	9	13	4	9	6261.035	6261.030	5.0
14	1	13	13	0	13	6262.945	6262.943	1.8
14	2	13	13	1	13	6275.990	6276.017	-27.0
21	2	19	20	3	18	6291.005	6291.002	3.0
21	3	19	20	3	18	6296.169	6296.166	3.8
21	3	19	20	2	18	6305.181	6305.188	-6.5
20	4	17	19	3	16	6352.429	6352.431	-1.7
11	7	5	10	6	4	6385.962	6385.966	-3.7
11	7	4	10	6	5	6386.026	6386.025	1.6
22	1	21	21	2	20	6393.957	6393.963	-5.9
22	2	21	21	2	20	6394.069	6394.072	-2.8
22	1	21	21	1	20	6394.163	6394.169	-6.7
22	2	21	21	1	20	6394.283	6394.278	5.0
21	3	18	20	4	17	6401.821	6401.819	2.1
15	5	11	14	4	10	6414.512	6414.517	-5.2
14	5	10	13	4	10	6436.110	6436.115	-5.0
21	4	18	20	4	17	6470.574	6470.566	7.6
14	5	9	13	4	10	6486.025	6486.021	3.3

J'	K_a'	K_c'	J"	K_a"	K_c"	ν_{obs}/MHz	ν_{calc}/MHz	$\Delta\nu$/kHz
23	0	23	22	1	22	6493.602	6493.590	11.4
23	1	23	22	0	22	6493.602	6493.593	8.8
10	8	3	9	7	2	6530.108	6530.109	-1.0
10	8	2	9	7	3	6530.108	6530.109	-1.2
13	6	8	12	5	7	6534.340	6534.329	11.0
13	6	7	12	5	7	6535.762	6535.772	-9.2
14	4	10	13	3	11	6541.504	6541.499	5.2
13	6	8	12	5	8	6545.820	6545.811	9.4
13	6	7	12	5	8	6547.252	6547.253	-0.9
22	2	20	21	3	19	6571.802	6571.804	-2.1
15	4	12	14	3	12	6578.348	6578.350	-2.6
21	4	18	20	3	17	6579.087	6579.087	0.0
22	3	20	21	2	19	6579.887	6579.887	0.2
16	5	12	15	4	11	6588.748	6588.758	-10.5
15	3	13	14	2	13	6609.388	6609.392	-3.7
16	3	13	15	2	13	6620.265	6620.259	6.0
23	1	22	22	2	21	6672.384	6672.380	4.0
23	2	22	22	1	21	6672.539	6672.546	-7.6
9	9	0	8	8	1	6672.641	6672.639	1.7
9	9	1	8	8	0	6672.641	6672.639	1.7
12	7	6	11	6	5	6696.226	6696.227	-1.2
12	7	5	11	6	6	6696.426	6696.426	0.1
22	3	19	21	4	18	6710.425	6710.419	5.5
16	5	11	15	4	11	6752.630	6752.622	8.1
15	5	11	14	4	11	6771.772	6771.780	-7.6
24	0	24	23	1	23	6772.020	6772.023	-2.6

J'	K_a'	K_c'	J"	K_a"	K_c"	ν_{obs}/MHz	ν_{calc}/MHz	$\Delta\nu$/kHz
24	1	24	23	0	23	6772.020	6772.024	-3.9
17	4	13	16	3	13	6778.790	6778.795	-4.2
22	4	19	21	3	18	6821.692	6821.692	-0.9
14	6	9	13	5	8	6829.217	6829.216	1.5
14	6	8	13	5	8	6832.745	6832.735	10.4
11	8	4	10	7	3	6841.852	6841.849	2.8
11	8	3	10	7	4	6841.852	6841.850	1.8
23	2	21	22	3	20	6851.448	6851.450	-1.8
14	6	9	13	5	9	6854.062	6854.076	-13.6
23	3	21	22	2	20	6856.005	6856.002	2.2
14	6	8	13	5	9	6857.596	6857.595	1.3
24	1	23	23	2	22	6950.771	6950.775	-3.8
24	2	23	23	1	22	6950.865	6950.862	2.9
10	9	2	9	8	1	6984.755	6984.760	-5.1
10	9	1	9	8	2	6984.755	6984.760	-5.1
19	5	15	18	4	14	6986.291	6986.293	-2.2
16	4	13	15	3	13	6986.542	6986.553	-11.0
13	7	7	12	6	6	7005.386	7005.378	7.9
13	7	6	12	6	7	7005.973	7005.972	1.5
25	0	25	24	1	24	7050.458	7050.455	3.4
25	1	25	24	0	24	7050.458	7050.455	2.7
15	6	10	14	5	9	7113.322	7113.306	16.1
16	5	12	15	4	12	7119.041	7119.036	4.6
15	6	9	14	5	9	7121.272	7121.270	2.1
12	8	5	11	7	4	7153.245	7153.234	10.6
12	8	4	11	7	5	7153.245	7153.239	6.2

J'	K_a'	K_c'	J"	K_a"	K_c"	ν_{obs}/MHz	ν_{calc}/MHz	$\Delta\nu$/kHz
15	6	9	14	5	10	7171.177	7171.177	0.7
21	5	17	20	4	16	7225.942	7225.933	8.2
25	1	24	24	2	23	7229.181	7229.161	19.8
25	2	24	24	1	23	7229.182	7229.207	-24.9
11	9	3	10	8	2	7296.784	7296.785	-0.6
11	9	2	10	8	3	7296.784	7296.785	-0.6
14	7	8	13	6	7	7312.935	7312.932	3.0
14	7	7	13	6	8	7314.530	7314.527	3.3
26	0	26	25	1	25	7328.894	7328.885	8.9
26	1	26	25	0	25	7328.894	7328.885	8.6
24	4	20	23	5	19	7329.285	7329.281	3.7
24	4	21	23	3	20	7339.109	7339.113	-4.0
16	6	11	15	5	10	7381.231	7381.245	-13.4
10	10	0	9	9	1	7439.221	7439.220	0.6
10	10	1	9	9	0	7439.221	7439.220	0.6
13	8	6	12	7	5	7464.141	7464.117	23.7
13	8	5	12	7	6	7464.141	7464.134	6.9
17	2	15	16	1	15	7471.132	7471.141	-9.3
16	6	11	15	5	11	7474.713	7474.710	3.7
16	6	10	15	5	11	7491.563	7491.573	-10.1
26	1	25	25	2	24	7507.538	7507.545	-7.3
26	2	25	25	1	24	7507.575	7507.568	7.0
17	3	15	16	2	15	7510.516	7510.505	10.7
23	5	19	22	4	18	7525.460	7525.442	17.4
27	0	27	26	1	26	7607.299	7607.314	-14.9
27	1	27	26	0	26	7607.299	7607.314	-15.0

J'	K_a'	K_c'	J"	K_a"	K_c"	ν_{obs}/MHz	ν_{calc}/MHz	$\Delta\nu$/kHz
12	9	4	11	8	3	7608.636	7608.638	-1.7
12	9	3	11	8	4	7608.636	7608.638	-1.7
15	7	9	14	6	8	7618.153	7618.146	6.8
17	6	12	16	5	11	7626.680	7626.671	8.2
11	10	2	10	9	1	7751.351	7751.357	-6.1
11	10	1	10	9	2	7751.351	7751.357	-6.1
14	8	7	13	7	6	7774.325	7774.322	3.4
14	8	6	13	7	7	7774.377	7774.378	-0.4
27	1	26	26	2	25	7785.949	7785.929	19.8
27	2	26	26	1	25	7785.949	7785.941	7.6
17	6	12	16	5	12	7790.551	7790.535	15.9
17	6	11	16	5	12	7824.156	7824.153	3.2
28	0	28	27	1	27	7885.738	7885.741	-3.9
28	1	28	27	0	27	7885.738	7885.741	-4.0
16	7	10	15	6	9	7919.859	7919.854	4.9
13	9	5	12	8	4	7920.235	7920.230	4.9
13	9	4	12	8	5	7920.235	7920.230	4.6
16	7	9	15	6	9	7920.886	7920.885	0.9
16	7	9	15	6	10	7928.847	7928.849	-2.0

Table S10: Assigned rotational transitions for the singly substituted ^{54}Fe isotopologue of the hetero-chiral isomer HET-1.

J'	K_a'	K_c'	J"	K_a"	K_c"	ν_{obs}/MHz	ν_{calc}/MHz	$\Delta\nu$/kHz
9	0	9	8	1	8	2585.833	2585.822	11.7

J'	K_a'	K_c'	J"	K_a"	K_c"	ν_{obs}/MHz	ν_{calc}/MHz	$\Delta\nu$/kHz
9	1	9	8	0	8	2611.612	2611.612	-0.2
10	0	10	9	1	9	2870.093	2870.095	-1.9
10	1	10	9	0	9	2884.559	2884.560	-0.7
10	1	9	9	2	8	2926.285	2926.279	5.4
11	1	11	10	0	10	3159.953	3159.940	13.6
11	1	10	10	2	9	3251.774	3251.784	-10.1
6	4	3	5	3	2	3467.149	3467.156	-7.2
6	4	2	5	3	3	3473.952	3473.951	1.0
5	5	1	4	4	0	3616.758	3616.747	11.6
5	5	0	4	4	1	3616.758	3616.760	-1.5
13	1	13	12	0	12	3714.477	3714.476	0.8
7	4	4	6	3	3	3769.581	3769.580	1.1
13	1	12	12	2	11	3861.925	3861.928	-2.5
14	0	14	13	1	13	3991.442	3991.436	6.1
14	1	13	13	2	12	4153.516	4153.516	0.4
7	5	3	6	4	2	4240.171	4240.175	-4.3
7	5	2	6	4	3	4240.771	4240.772	-1.4
6	6	1	5	5	0	4385.770	4385.770	0.4
6	6	0	5	5	1	4385.770	4385.770	-0.2
16	0	16	15	1	15	4549.322	4549.329	-6.3
16	1	16	15	0	15	4549.647	4549.657	-9.9
8	5	4	7	4	3	4550.160	4550.153	7.0
8	5	3	7	4	4	4552.345	4552.343	1.5
7	6	2	6	5	1	4697.943	4697.936	7.5
7	6	1	6	5	2	4697.943	4697.943	0.3
10	4	6	9	3	7	4790.867	4790.866	0.7

J'	K_a'	K_c'	J"	K_a"	K_c"	ν_{obs}/MHz	ν_{calc}/MHz	$\Delta\nu$/kHz
9	5	5	8	4	4	4857.362	4857.360	2.3
9	5	4	8	4	5	4863.904	4863.904	0.1
7	7	1	6	6	0	5154.761	5154.767	-6.2
7	7	0	6	6	1	5154.761	5154.767	-6.2
10	5	5	9	4	6	5176.356	5176.346	10.1
9	6	4	8	5	3	5321.151	5321.149	2.6
9	6	3	8	5	4	5321.336	5321.337	-0.7
8	8	0	7	7	1	5923.741	5923.745	-4.0
8	8	1	7	7	0	5923.741	5923.745	-4.0
9	8	2	8	7	1	6236.003	6235.984	18.5
9	8	1	8	7	2	6236.003	6235.984	18.5
11	7	5	10	6	4	6401.900	6401.905	-5.1
11	7	4	10	6	5	6401.953	6401.960	-6.8
10	8	3	9	7	2	6548.096	6548.097	-1.0
10	8	2	9	7	3	6548.096	6548.097	-1.2
9	9	0	8	8	1	6692.702	6692.702	0.3
9	9	1	8	8	0	6692.702	6692.702	0.3
11	8	4	10	7	3	6859.978	6859.981	-2.4
11	8	3	10	7	4	6859.978	6859.982	-3.4
10	9	1	9	8	2	7004.959	7004.961	-1.1
10	9	2	9	8	1	7004.959	7004.961	-1.1
12	8	5	11	7	4	7171.512	7171.515	-2.4
12	8	4	11	7	5	7171.512	7171.519	-6.6
11	9	3	10	8	2	7317.125	7317.125	0.4
11	9	2	10	8	3	7317.125	7317.125	0.4
10	10	0	9	9	1	7461.631	7461.635	-3.5

J'	K_a'	K_c'	J"	K_a"	K_c"	ν_{obs}/MHz	ν_{calc}/MHz	$\Delta\nu$/kHz
10	10	1	9	9	0	7461.631	7461.635	-3.5
